# Investigating Developmental and Epileptic Encephalopathy Using *Drosophila melanogaster*

**DOI:** 10.3390/ijms21176442

**Published:** 2020-09-03

**Authors:** Akari Takai, Masamitsu Yamaguchi, Hideki Yoshida, Tomohiro Chiyonobu

**Affiliations:** 1 Department of Pediatrics, Graduate School of Medical Science, Kyoto Prefectural University of Medicine, Kyoto 602-8566, Japan; takaiaka@koto.kpu-m.ac.jp; 2 Department of Applied Biology, Kyoto Institute of Technology, Matsugasaki, Sakyo-ku, Kyoto 603-8585, Japan; myamaguc8@gmail.com (M.Y.); hyoshida@kit.ac.jp (H.Y.); 3 Kansai Gakken Laboratory, Kankyo Eisei Yakuhin Co. Ltd., Kyoto 619-0237, Japan

**Keywords:** developmental and epileptic encephalopathies, *Drosophila melanogaster*, early infantile epileptic encephalopathy, bang-sensitivity, genetic screening, drug screening

## Abstract

Developmental and epileptic encephalopathies (DEEs) are the spectrum of severe epilepsies characterized by early-onset, refractory seizures occurring in the context of developmental regression or plateauing. Early infantile epileptic encephalopathy (EIEE) is one of the earliest forms of DEE, manifesting as frequent epileptic spasms and characteristic electroencephalogram findings in early infancy. In recent years, next-generation sequencing approaches have identified a number of monogenic determinants underlying DEE. In the case of EIEE, 85 genes have been registered in Online Mendelian Inheritance in Man as causative genes. Model organisms are indispensable tools for understanding the in vivo roles of the newly identified causative genes. In this review, we first present an overview of epilepsy and its genetic etiology, especially focusing on EIEE and then briefly summarize epilepsy research using animal and patient-derived induced pluripotent stem cell (iPSC) models. The *Drosophila* model, which is characterized by easy gene manipulation, a short generation time, low cost and fewer ethical restrictions when designing experiments, is optimal for understanding the genetics of DEE. We therefore highlight studies with *Drosophila* models for EIEE and discuss the future development of their practical use.

## 1. Introduction

Epilepsy is defined as a disorder of the brain characterized by an enduring predisposition to generate epileptic seizures and by the neurobiologic, cognitive, psychological and social consequences of this condition. It is one of the most common diseases, the third leading contributor to the global burden of disease for neuronal disorders and affects 65 million people worldwide. Both clinically and etiologically, epilepsy is a very diverse disease. It can be classified into several epilepsy syndromes based on clinical features [1] but each syndrome ranges from self-limiting to drug-resistance. Approximately one-third of patients have difficulties to control seizures with anti-seizure drugs (ASDs). In particular, children with a group of epilepsy collectively referred to as ‘developmental and epileptic encephalopathy (DEE)’ have not only intractable seizures but also serious problems in psychomotor development, which poses a major lifelong problem. A deeper understanding of mechanisms is required to overcome such difficult diseases and various animal models are used for this purpose. This is particularly the case for monogenic epilepsies. In this review, we give an overview of animal models for epilepsy, mainly in *Drosophila*.

## 2. Pathophysiology of Epilepsy

**Figure 1:**
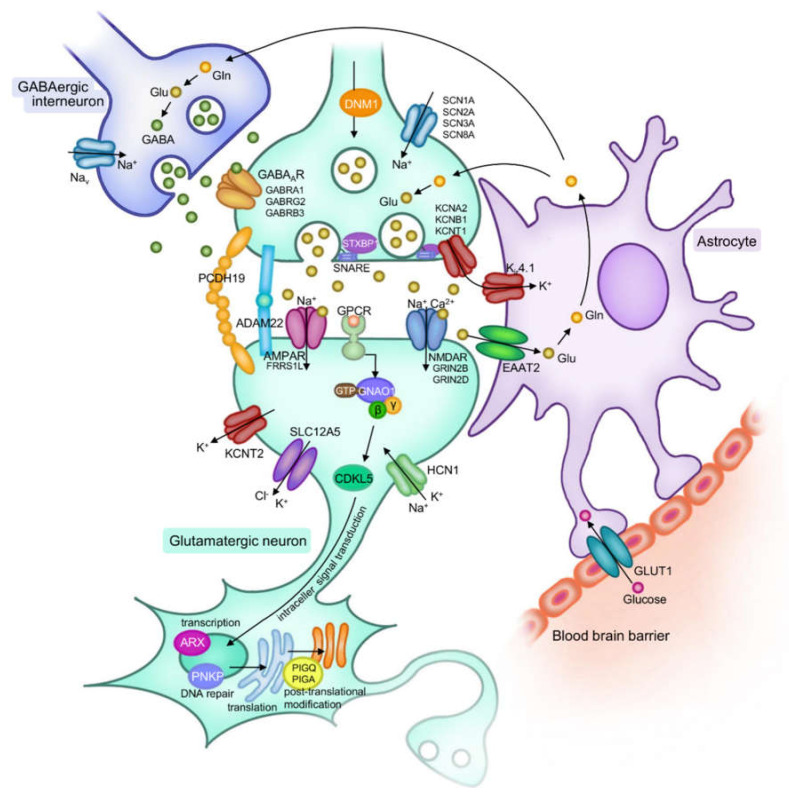
Schematic model depicting main functions associated with monogenic developmental and epileptic encephalopathies (DEEs).

An epileptic seizure is defined as the transient occurrence of signs and/or symptoms due to abnormal excessive or synchronous neuronal activity in the brain. This excessive neuronal excitability can occur not only in the dysfunction of excitatory/inhibitory neurons but also in glial abnormalities [2,3]. Many pathophysiological processes have been described to cause monogenic epilepsies, including structural or functional changes in ion channels, neurotransmission, transporters, inter-neuronal connectivity, intra-neuronal signal transduction, transcription and translation/post translational modification (Figure 1, Table 1). Importantly, these pathologies can also cause neurological complications such as intellectual disability, neurodevelopmental disorders and psychiatric disorders.

Pathogenic variants affecting ion channels, neurotransmission, transporters, inter-neuronal connectivity, intra-neuronal signal transduction, transcription and translation/post translational modification are identified in DEE patients.

## 3. Developmental and Epileptic Encephalopathy (DEE)

### 3.1. Overview of DEEs

DEEs, the spectrum of severe forms of epilepsies, are characterized by early-onset, refractory seizures that also occur in the context of developmental regression or plateauing. DEEs encompass several clinically definable epilepsy syndromes such as early infantile epileptic encephalopathy (EIEE; also known as Ohtahara syndrome), West syndrome (WS), epilepsy of infancy with migrating focal seizures (EIMFS), Dravet syndrome (DS) and Lennox-Gastaut syndrome (LGS). However, some patients do not fall into any epilepsy syndrome and are diagnosed with unclassified DEE. With advances in sequencing methods, many of the patients with DEE now have an identifiable molecular genetic basis, including pathogenic copy number variants (CNVs) and monogenic mutations [4,5]. Suppressing severe epileptic activity by anti-seizure treatment may play a role in improving developmental progress; therefore, active management for seizures is required. On the other hand, we must understand that the genetic etiology itself influences the development, implying that the development of precision therapies is essential for the devastating consequences of DEEs.

### 3.2. Characteristics of Monogenic DEE

Growing numbers of DEE-causing genes have been identified by next-generation sequencing (NGS). Taking EIEE as an example, as of 30 April 2020, as many as 85 genes have been registered in Online Mendelian Inheritance in Man (OMIM) as causative genes (Table 1). EIEE is one of the earliest forms of DEE, manifesting as frequent epileptic spasms and characteristic electroencephalogram (EEG) findings (suppression-burst pattern) in early infancy. Almost all patients subsequently present with severe psychomotor retardation. The identification of DEE as a monogenic disorder is increasing and model organisms are indispensable tools for understanding the function of new causative genes. The functions and clinical features of the major EIEE-causing genes are outlined below.

Pathogenic variants in genes encoding brain-expressing ion channel components are the most frequent cause of DEE. *SCN1A* encodes voltage-gated sodium channel 1.1 (Na_V_1.1), which mainly makes the sodium current in the inhibitory GABAergic neurons. Heterozygous loss-of-function variants of *SCN1A* are identified in approximately 80% of patients with Dravet syndrome, which is characterized by fever-induced status epilepticus, refractory myoclonic and absence seizures, ataxia, intellectual disability and autistic features [6]. Importantly, the pathogenic *SCN1A* variants have a wide range of phenotypes, which may be also identified in patients with other types of DEE, such as EIEE or milder forms of epilepsy such as genetic epilepsy with febrile seizures plus (GEFS+) [7]. On the other hand, *SCN2A* and *SCN8A* play essential roles in the excitability of glutamatergic neurons and most DEE-associated variants of both these genes are missense with gain-of-function [8,9,10]. Pathogenic variants of voltage-gated potassium channel genes, such as *KCNA2*, *KCNB1* and *KCNQ2*, were also identified in patients with EIEE [11,12]. *KCNT1*, encoding a sodium-activated potassium channel, was identified as the major causative gene for EIMFS, a rare DEE characterized by refractory migrating focal seizures beginning within 6 months [13].

Defects in genes encoding components of neurotransmitter receptors are also identified in DEE patients. Recently, many forms of DEE were found to be associated with variants in an increasing number of genes encoding GABA_A_ receptor subunits [14]. GABA_A_ receptors are composed of heteropentamers with different subunit combinations and function as ligand-gated anion channels. Loss-of-function variants mainly cause GABAergic disinhibition as the main disease mechanism. In contrast to GABA, glutamate is the major excitatory neurotransmitter and pathogenic variants of genes encoding glutamate-activated receptor subunits are also associated with DEEs. For example, *GRIN2B* gain-of-function variants have been identified as a cause of WS, a DEE presenting with clusters of infantile spasms and a characteristic EEG pattern called hypsarrhythmia [15].

Solute carriers (SLCs) are the family of transmembrane transporters that mediate the exchange of numerous substances, such as ions, nutrients and metabolites, across biological membranes. Several SLC genes expressed in the brain have been reported as a monogenic cause of DEEs. Among them, *SLC2A1* is an important causative gene from the viewpoint of therapeutic decision-making [16]. *SLC2A1* encodes GLUT1, the most prominent glucose transporter of the human brain and haploinsufficiency of *SLC2A1* causes GLUT1 deficiency syndrome (GLUT1-DS), characterized by varying degrees of intellectual disability, epilepsy and movement disorders. It is well known that the ketogenic diet is an effective treatment for GLUT1-DS, as it bypasses glucose metabolism [17].

Disruption of synaptic exocytosis or membrane trafficking is one of the major pathologies of DEEs. Syntaxin-binding protein 1 (STXBP1, also known as MUNC18-1) plays an important role in synapse vesicle docking and fusion in concert with both vesicle-associated and target-associated soluble N-ethylmaleimide-sensitive factor attachment protein receptor (SNARE) proteins. Initially, haploinsufficiency of *STXBP1* was reported as the cause of EIEE [18]. Importantly, subsequent studies broadened the phenotypic spectrum of STXBP1 encephalopathy to WS, DS, unclassified DEE and intellectual disability without epilepsy [19].

Abnormalities of cell-adhesion molecules are also involved in the development of DEEs. The X-chromosome gene *PCDH19* encodes the cell-adhesion protein protocadherin-19 and is responsible for a female-limited epilepsy with intellectual disability and autistic features [20]. Of note, hemizygous males are generally unaffected. Although the cellular and molecular mechanisms that lead to epilepsy in females are not completely understood, the mosaic state of cells expressing either the normal or mutant allele caused by random X-inactivation is thought to drive the pathology [21].

The *nonerhythrocytic α-spectrin-1* (*SPTAN1*) gene encodes cytoskeletal protein αII spectrin, which plays an important role in dendritic and axonal development and synaptogenesis. The majority of pathological variants found in EIEE patients reside in the last 2 spectrin repeats in the C-terminal region required for the formation of α/β spectrin heterotetramers and patient-derived neurons exhibited aggregation of spectrin complexes, suggesting a dominant-negative mechanism of *SPTAN1* variants in EIEE [22].

Abnormalities of several intracellular signaling molecules and transcription factors have been reported as the cause of DEEs. Among this group of genes, pathological variants in *CDKL5* (cyclin-dependent kinase like 5) are most frequently identified in DEE patients. CDKL5 belongs to the serine-threonine kinase family and is widely distributed in the human body. The highest expression levels are in the peri- and postnatal stages of the nervous system, suggesting an important role in the process of brain development [23]. Recent studies demonstrated that CDKL5 regulates axon outgrowth, dendritic morphogenesis and synapse formation [24]. The first identified EIEE-causing gene was *ARX*, encoding the transcription factor Aristaless-Related Homeobox [25]. Expansions in the first and second poly-alanine tracts in ARX cause a spectrum of disorders, from EIEE to non-syndromic mental retardation [26]. *ARX* regulates the transcription of genes involved in GABAergic interneuron development. After the discovery of this disease, a new concept of ‘interneuronopathy’ was proposed as a pathological condition of epilepsy [27].

Recently, genetic defects in the glycosylphosphatidylinositol (GPI) biosynthesis pathway have been identified as the causes of disorders with a wide range of symptoms, including DEEs [28]. GPI is a glycolipid that anchors 150 or more kinds of proteins to the human cell surface. More than 20 genes are involved in the biosynthesis and remodeling of GPI-anchored proteins. Among them, pathogenic variants of *PIGA* have been most frequently identified in EIEE patients [29,30]. Pathological variants of other phosphatidyl inositol glycan (PIG) genes have also been identified in patients with EIEE or WS [31,32,33].

## 4. Animal and Patient-Derived iPSC Models for Epilepsy Research

Although NGS of the entire genome of patients with epilepsy symptoms has identified a number of genes related to epilepsy, as described above, in vivo functions of these genes are not well known. Furthermore, due to ethical restrictions, clinical studies involving epileptogenesis and ictogenesis are difficult to carry out in humans. Animal models and patient-derived induced pluripotent stem cell (iPSC) models are therefore required to effectively advance epilepsy research [34,35,36,37]. Recent progress in gene editing technologies, such as the CRISPR/Cas9 system, has further facilitated the development of animal and iPSC models for epilepsy syndromes. Each of the models has advantages and disadvantages. Among these model organisms, we compared *Drosophila* (*Drosophila melanogaster*), nematode (*Caenorhabditis elegans*) [38], zebrafish (*Danio rerio*), mouse (*Mus musculus*) [39] and human iPSC [40] (Table 2). Due to the simplicity of the genome and availability of many mutants and RNAi lines, *Drosophila* and nematodes are more suitable to perform genome-wide genetic screening than other models. Mice and zebrafish contain more neuronal cells and are more suitable for studies of complex behavior than *Drosophila* and nematodes. However, fewer ethical concerns when conducting experiments is another advantage for *Drosophila* and nematode models. Shorter generation times are also an advantage for these two models. Patient-derived iPSC models are suitable for epilepsy studies in the context of the unique genetic constellation of the individual that is otherwise difficult to assess with animal models. In the next section, animal models for epilepsy other than the *Drosophila* model are summarized, especially focusing on genetic models.

### 4.1. Rodent Epilepsy Models

Rodent models have been widely used for examining the etiology and pathogenesis of epilepsy [41]. In general, two types of rodent models are known for epilepsy, non-genetic and genetic models. In non-genetic models, focal application of tungstic acid, cobalt, acetylcholine, strychnine or picrotoxin to rodents, including mice and rats, can induce seizures [42,43]. These acquired epilepsy rodent models have been useful to understand the relationship between EEG events and the underlying firing activity of individual neurons [35]. The so-called kindled rodent models for epilepsy were developed by repetitive electrical or chemical stimulation of the rodent brain to produce kindling conditions, in which the threshold for electrically stimulated seizures decreases and spontaneous seizures can develop [44]. These kindled rodents representing models of complex partial seizures have been used to establish seizure thresholds and evaluate possible therapies [35]. Administration of pilocarpine or kainic acid to rodents can similarly induce acute epilepsy together with recurrent spontaneous seizures. As neuronal damage and synaptic reorganization, which is axonal sprouting, are generated in these rodent models, they are often used as temporal lobe epilepsy (TLE) models [35]. These rodent models have been used to investigate mechanisms and to search for biomarkers of epileptogenesis or to evaluate novel candidate drugs that were identified by high-throughput screening. However, differences in the genetic background among heterogeneous populations and even inbred strains should be considered when interpreting the data [45,46]. Indeed, some strains of mice are known to be more resistant to kainic acid than others [47]. It is also known that there are differences between mice and rats in several aspects of the epileptogenic process [35]. Furthermore, susceptibility of different strains of mice to seizures does not always correlate with underlying factors for epilepsy such as neuron loss and mossy fiber sprouting [48].

The well-known classical genetic models for epilepsy in mice are Tottering mice and a more severe allele, Leaner mice, carrying spontaneous mutations in the *α1a voltage-sensitive calcium channel 1A* (*α1A*) gene [49,50]. Expression of the *α1A* gene is observed in the central nervous system (CNS), with prominent and uniform expression in the cerebellum. These mice exhibit absence-like seizures and intermittent focal seizures that are similar to human seizure disorders, suggesting the importance of voltage-gated calcium channels in understanding human seizure disorders [35]. Transgenic and knockout mouse technologies enabled the development of a variety of genetic mouse models as epileptic models with spontaneous recurrent seizures or seizure susceptible models with a reduced threshold for acute induction of seizures. There are a number of transgenic mice reproducing specific human conditions such as type I lissencephaly targeting of the *Lis1* gene [51], tuberous sclerosis complex (TSC) targeting of the *TSC1* gene [52] or DS targeting of the *SCN1A* gene (Table 1) [53]. Homozygous null *SCN1A* mutant mice exhibited ataxia and died on postnatal day 15. Heterozygous *SCN1A* mutant mice exhibited spontaneous seizures and sporadic death after postnatal day 21. In the inhibitory interneurons of heterozygous *SCN1A* mutant mice, the sodium current density was reduced. However, this was not the case in their excitatory pyramidal neurons. These observations suggest that reduced sodium currents in GABAergic inhibitory interneurons in the heterozygous *SCN1A* mutant mice cause the hyperexcitability, leading to epilepsy in patients with DS [53]. These mouse models helped to identify the underlying defects leading to epileptic conditions and to advance therapeutic interventions, such as rapamycin, designed to target specific signaling pathways associated with TSC and the potential clonazepam-mediated rescue of autism-related co-morbidities in DS [35]. Rodent models showing the role of glia-induced hyperexcitability in epilepsy has also been reported. For example, astrocyte-specific deletion of K_ir_4.1, an inwardly rectifying potassium channel, impairs potassium ion transfer and glutamate uptake by astrocytes, causing seizures, ataxia and premature death in mice [54]. In addition, down-regulation of astrocytic Kir4.1 in some brain regions were reported in rodent epilepsy models [55,56]. These findings suggest that disruption of spatial potassium-buffering function of astrocytes is involved in the development of epilepsy.

### 4.2. Zebrafish Epilepsy Models

Zebrafish is another well-established vertebrate model for examining the function of the genes related to neurodevelopmental disorders, including epilepsy [57,58]. External development of transparent embryos makes it easy to visualize the nervous system during development (Table 2). Relatively large progenies are advantageous for carrying out high-throughput pharmacological screens to identify candidate substances for therapy based on simple behavioral phenotypes. Of note, zebrafish membranes are generally permeable to substances placed in the bathing medium, which makes them suitable for drug screening [35]. For epilepsy studies in particular, administration of the GABA-A antagonist, pentylenetetrazol (PTZ) to wild-type larvae can induce robust, seizure-like behaviors, such as rapid burst-like and circling movements, enabling quantification of seizure susceptibility [59]. Zebrafish mutants of epilepsy-associated genes exhibit spontaneous seizures and increased sensitivity to PTZ [57,60,61]. Furthermore, both drug-induced and spontaneous locomotor seizures are associated with electrographic seizures [59,60] and can be easily evaluated in high-throughput assays, making zebrafish a useful model in drug screening for epilepsy syndromes [60,62].

The *scn1lab* gene is a zebrafish orthologue of human *SCN1A* (Table 1) [60]. Homozygous *scn1lab* mutants exhibit spontaneous seizures beginning at 4 days post fertilization, accompanied by electrographic seizures [60]. High-throughput screening using these mutants identified clemizole, an antihistamine, as suppressors of both seizure-like behaviors and electrographic seizures [60]. Clemizole has activity at 5-HT2A and 5-HT2B receptors [63]. In the zebrafish *scn1lab* mutants and morphants established by the injection of morpholino oligonucleotides, fenfluramine, an inducer of serotonin (5-hydroxytrypamine, 5-HT) release, was reported to reduce seizure activity [64,65,66]. This suggests that a serotonergic pathway is responsible for anti-epileptic activity. Using these *scn1lab* mutants, 5-HT receptor agonists and 5-HT-modulating compounds, such as lorcaserin and trazodone, were found to repress seizure activity [63,66,67]. Of note, Fenfluramine and lorcaserin improved seizures in individuals with DS [63,67,68], suggesting conservation of pharmacological pathways between humans and zebrafish. Importantly, chronic fenfluramine administration not only suppressed seizures but also completely restored dendritic arbor numbers of GABAergic neurons to normal in *scn1ab^mut/mut^* zebrafish, indicating the potential of fenfluramine as a disease-modifying drug [69]. In addition, zebrafish mutants of *stxbp1b*, an orthologue of human *STXBP1* (Table 1), exhibit electrographic seizures at baseline. These mutants may also be a useful model to examine *STXBP1* encephalopathy [61]. Recently, a zebrafish model for *CACNA1A*-related epilepsy was also reported (Table 1) [70]. This model was shown to be able to evaluate the effects of various ASDs, indicating that it can be applied to new drug screening.

### 4.3. Nematode Epilepsy Models

*C. elegans*, a non-parasitic nematode, is a well-established invertebrate model for investigating the development and function of the nervous system. Orthologues of approximately 65% identity to human disease-related genes exist in *C. elegans* (Table 2). The nematode contains 302 neurons in adult hermaphrodites (383 in males) that can be divided into 118 neuronal cells and 56 glial cells (Table 2). Neurotransmitters in *C. elegans* comprise glutamate, dopamine, serotonin, GABA and acetylcholine but not epinephrine, norepinephrine or histamine. In contrast to other animal models, voltage-gated sodium channels are missing in *C. elegans* [71]. *C. elegans* models have several unique properties for studying epilepsy that may be complementary to vertebrate models. Nematodes are transparent and neurons can be visualized in vivo using fluorescent markers. Moreover, using live calcium imaging, the temporal patterns of neuronal activity can be linked to behavioral phenotypes in *C. elegans* [72].

Electrical shock or increasing the ambient temperature can induce seizures in wild-type *C. elegans* [73,74]. Convulsions in *C. elegans* can be induced in genetic models carrying mutations in the *lis-1* gene having defective GABA transmission. PTZ treatment and RNAi mediated knockdown of the genes related to the LIS1 pathway, such as *NUD-1 and NUD-2 and DHC-1, CDK-5 or CDKA-1,* can also induce convulsions [73]. In these genetic models, the resulting seizures in *C. elegans* mean repeated contractions in either the dorsal or ventral direction [73].

## 5. *Drosophila* Epilepsy Models

*Drosophila melanogaster* is a good model organism for studying genetics, developmental biology and neurobiology [75]. *Drosophila* is small, inexpensive to maintain and easy to manipulate under standard laboratory conditions. *Drosophila* has a short life span of 10 days and produces a large number of offspring by laying 100 eggs per day, which facilitates statistical analyses (Table 2). The *Drosophila* genome database and information on genetic resources are conveniently available in the FlyBase (https://flybase.org/). A number of mutants and RNAi lines are available from stock centers such as the Bloomington Drosophila Stock Center (https://bdsc.indiana.edu/), Kyoto Stock Center (http://www.dgrc.kit.ac.jp/) and Vienna Drosophila Resource Center (https://stockcenter.vdrc.at/control/main) [75]. Furthermore, *Drosophila* exhibit complex behaviors, including social activity, learning and memory and courtship. In addition, there are fewer ethical concerns when designing experiments with *Drosophila* because insects are not included in animal laws.

Nervous systems, particularly in excitable membrane components, are similar between *Drosophila* and humans [76]. Voltage-gated and ligand-gated signaling molecules, such as Na+, K+ and Ca^2+^ channels and glutamate, acetylcholine and GABA transmitter receptors are highly conserved between the two species [76]. Although the *Drosophila* CNS is organized as a ganglionic structure with synaptic neuropilar regions instead of the human cortex consisting of synaptic layers, electrical shock of sufficient intensity delivered to the *Drosophila* adult brain can induce neuronal spiking activity that is seizure-like in appearance [76]. As described above, many human genes responsible for EIEE, including those related to ion channels, neurotransmitter receptors, solute carrier family, synaptic vesicle release, membrane trafficking, cell adhesion, cytoskeleton, intracellular signal transduction, transcription, translation, post-translational modification and epigenetics, are conserved in *Drosophila* (Table 1). *Drosophila* has thus become an attractive model organism to examine human epilepsy with the purpose of investigating in vivo functions of responsible genes, identifying novel biomarkers and performing in vivo drug screening [77].

### 5.1. Drosophila Models for EIEE

*Drosophila* models targeting EIEE-associated genes and in vivo studies on the EIEE-associated genes in *Drosophila* are summarized in the following sections.

#### 5.1.1. *Drosophila* Models Targeting Voltage-Gated Sodium Channels (Nav)

Extensive screens for temperature-sensitive mutations causing paralysis at a restrictive temperature identified the *paralysis* (*para*) mutation (*para^ts1^*) [78,79] and several other *para* alleles exhibiting heat-induced paralysis. However, unlike the epilepsy-causing sodium channel mutations in humans, these *Drosophila* mutants do not exhibit temperature-sensitive seizure phenotypes [79]. However, later, one of the bang-sensitive (BS) mutants, *bang senseless* (*para^bss1^*), was found to be highly sensitive to seizures, exhibiting seizure-like behaviors and paralysis following mechanical, electrical or visual stimulation. [80,81,82].

In the *Drosophila* genome, *para* is the only gene that encodes voltage-gated sodium channel α-subunits and is a single orthologue of the human *SCN1A*, *SCN2A, SCN3A, SCN4A, SCN5A, SCN7A, SCN8A, SCN9A, SCN10A and SCN11A* genes. Among them, the *SCN1A*, *SCN2A, SCN3A* and *SCN8A* genes are associated with EIEE (Table 1). The *Drosophila para* transcript produces a variety of sodium channels by alternative splicing [83,84]. *Drosophila* para (Nav) sodium channel structure is similar to that of human sodium channels. Domains important for channel function are highly conserved between the two species [85]. Sodium channel α-subunits are composed of four homologous domains (domain I–IV) and each of them contains six transmembrane segments (S1–S6). The ion pore is formed centrally by the collective organization of S5–S6 segments from each domain. Surrounding the ion pore, the four voltage sensors are composed of S1–S4 segments from each domain. A “paddle motif,” S3b–S4 helix–turn–helix motif that is responsible for activation of the voltage sensors and opening and closing of the pore, is considered to be important for the action of each voltage sensor [86,87,88]. Detailed biochemical and physical analyses suggested that the voltage sensor paddles of domains I–III drive channel activation, whereas the paddle of domain IV drives channel inactivation [87]. The *para^bss1^* is a gain-of-function mutant carrying a single amino acid substitution (leu to phe) at amino acid position 1699 within the hydrophobic S3b membrane-spanning segment of homology domain IV [76,82].

**Figure 2:**
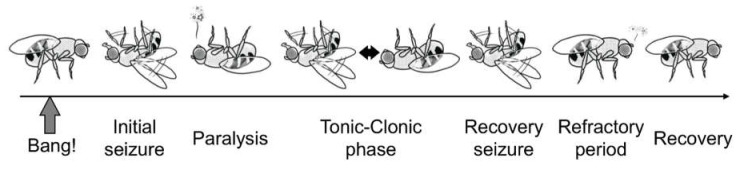
*Drosophila* “bang-sensitive” seizure.

The *para^bss1^* flies demonstrate common behaviors similar to wild type flies under normal conditions. A mechanical shock, such as a tap of the culture vial or brief vortex mixing termed “bang,” can induce abnormal behavior in *para^bss1^* mutants. The induced behavioral phenotype can be divided into six distinguishable phases (Figure 2) [76]. (1) Initial seizure—flies exhibit leg shaking, abdominal muscle contractions, wing flapping and scissoring and proboscis extensions for several seconds. (2) Paralysis—flies are immobile and unresponsive to mechanical stimulus. (3) Tonic-clonic phase—flies are quiescent, resembling a tonic phase that is broken up by multiple bouts of clonus-like activity. (4) Recovery seizure—similar to the initial seizure and clonus-like activity. (5) Refractory period—flies are behaviorally normal but cannot be induced to exhibit further seizures. (6) Recovery—flies are completely recovered and regain bang sensitivity. In addition to this bang-induced seizure-like activity, the *para^bss1^* flies have a lower threshold for electric shock-induced seizures [76,82]. In addition, a convenient low-cost method to monitor seizure-like activity of *Drosophila* has been developed. This method utilizes a web-cam to capture images, which are processed using software to track the distance moved, the average velocity of movement and the duration of movement during a specified time-span [89].

Figure shows a typical behavior of *para^bss1^* mutant subjected to a mechanical shock (brief vortex mixing termed “Bang!”). The induced behavioral phenotype can be divided into six distinguishable phases as (1) Initial seizure, (2) Paralysis, (3) Tonic-clonic phase, (4) Recovery seizure, (5) Refractory period and (6) Recovery.

The *SCN1A^K1270T^* mutation in *SCN1A* was identified in GEFS+ patients [90]. The *SCN1A^S1231R^* mutation is another missense mutation identified in DS patients [91]. These epilepsy-causing *SCN1A* mutations are found in transmembrane segments S1 and S2 of domain III in the sodium channels [84]. The *Drosophila para* gene was edited using the CRISPR-cas9 system to produce DS models carrying the *SCN1A^S1231R^* mutation and GEFS+ models carrying the *SCN1A^K1270T^* mutation in the *para* gene [84]. In contrast to the *Drosophila para* mutants identified in forward genetics screens, both of these *Drosophila* DS and GEFS+ models exhibited heat-induced seizures [92,93]. In detail, they exhibited loss of standing posture, followed by continuous movement of the legs, wings or abdomen in the fallen flies after heating. In addition, the DS model flies were more sensitive to the heat-induced seizures than the GEFS+ model flies. This phenotype is consistent with greater severity of DS over GEFS+ in humans [92,93]. Of note, flies that are mutually heterozygous for the DS and GEFS+ mutations are less sensitive to heat-induced seizures than the DS and GEFS+ homozygotes. These observations suggest that the underlying mechanisms of these two mutations are in opposite directions. The GEFS+ model flies have a lower threshold to elicit sodium current, resulting in a hyperpolarizing shift in the voltage dependence of persistent sodium current deactivation in the GABAergic neurons, increasing sodium currents over a wider voltage range [93]. In contrast, the DS model flies have a reduction of the sodium current and increase in the threshold to elicit sodium current, causing a depolarizing shift in the voltage-dependent deactivation of the persistent sodium current [92]. Both model flies exhibit reduced excitability in inhibitory GABAergic neurons, contributing to heat-induced seizure phenotypes [84]. In addition to *SCN1A*, the other sodium channel α-subunit genes, such as *SCN2A, SCN3A and SCN8A,* are also associated with EIEE, as described above (Table 1). Therefore, by taking a similar approach to *SCN1A*, *Drosophila* models targeting the *para* gene will be useful to examine EIEE associating mutations of these human genes.

The *SCN4A^G1306V^* mutation in the human *SCN4A* gene was identified in paramyotonia congenital, an autosomal dominant disorder in which myotonia can be elicited by exercise or cold temperatures [94]. The *Drosophila para^G1517R^* mutation corresponding to this mutation exhibits a dominant cold-sensitive paralytic phenotype. This is another example of a *Drosophila* model targeting the sodium channel.

The human *SCN1B* gene encodes the voltage-gated sodium channel β1 and β1B non-pore-forming subunits (Table 1) [95]. Originally, β subunits were characterized as auxiliary subunits for the sodium channel but they are now known to have a number of functions, including regulating channel assembly and gating and are involved in diverse and essential roles in multiple tissues. *SCN1B* is associated with EIEE (Table 1) [95]. Although *Drosophila* TipE and its paralogue TEH1-4 have less homology to human *SCN1B,* they may be functional homologues in *Drosophila* [96] (Table 1). *TipE* is involved in the cellular response to heat, male courtship behavior and regulation of sodium ion transport. Mutants of *TipE* are paralyzed at high temperatures and quickly recover when the temperature decreases [97]. The double mutant combination with *para^ts1^* results in action potential failure in recordings from larval motor neurons at a temperature at which either *para^ts1^* or *tipE* mutant alone exhibits normal nerve conduction [98], suggesting a cooperative role for the two genes. The *TipE* mutants may therefore function as good models to examine the biological roles of *SCN1B* in DEE pathogenesis.

#### 5.1.2. *Drosophila* Models Targeting Voltage-Gated Potassium Channel (Kv)

The *Drosophila Shaker* (*Sh*) gene encodes the α subunit of a voltage-gated potassium channel (Kv1), playing a role in maintaining electrical excitability in neurons and muscle cells and in regulation of neurotransmitter release at the synapse [99]. *Sh* has high homology with the human *KCNA1*, *KCNA2 and KCNA3* genes (Table 1). As described above, mutations in *KCNA2* are associated with EIEE [100], whereas mutations in the *KCNA1* gene are associated with a variety of movement disorders [101]. Detailed phenotypic analyses of *Sh* mutants revealed that it is involved in several processes, including mating behavior, sex discrimination, proboscis extension reflex and regulation of synaptic activity [102].

The *Drosophila Shaker cognate b* (*Shab*) gene encodes a member of the Sh family, the α subunit of a delayed rectifier potassium channel (Kv2). The channel regulates excitability in neurons and muscles and transmitter release. *Shab* has high homology with the human *KCNB1* and *KCNB2* genes (Table 1). As described above, mutations in *KCNB1* are associated with EIEE [103]. Phenotypic analyses of *Shab* mutants suggested its involvement in several processes, including larval locomotive behavior, positive regulation of circadian sleep/wake rhythm, sleep and regulation of synaptic activity. Detailed studies with *Drosophila* models revealed that Sh and Shab play distinct roles in frequency-dependent regulation of nerve terminal excitability and synaptic transmission [104]. These *Drosophila* models may be useful to study epilepsy syndromes related to potassium channels.

The *Drosophila KCNQ* gene encodes another voltage-gated potassium channel α subunit involved in several biological processes, including cardiac muscle contraction, embryonic development and regulation of the heart rate [105]. *KCNQ* is orthologous to several human genes, including *KCNQ1* to *5*. Among them, *KCNQ2* is related to EIEE, as described above [106]. Further analyses may be necessary to evaluate the suitability of *Drosophila* models targeting *KCNQ* in studies of *KCNQ2*-related EIEE. In addition, *Drosophila slowpoke 2* (*SLO2*) encodes a sodium activated and/or calcium-activated potassium channel. Its human homologues, such as *KCNT1* and *KCNT2,* are associated with EIEE, although few studies have been performed with *Drosophila* models targeting *SLO2* (Table 1).

#### 5.1.3. *Drosophila* Models Targeting Hyperpolarization-Activated Cyclic Nucleotide-Gated (HCN) Channels

HCN channels are low-threshold, voltage-gated ion channels. They are normally activated at negative potentials. The *Drosophila Ih* channel gene encodes a low-threshold, voltage-gated ion channel that influences excitatory postsynaptic potential kinetics and integration. Administration of the drug ZD7288 to wild-type *Drosophila* larvae inhibited the presynaptic HCN channel activity and reduced the increase in neurotransmitter release at motor neuron terminals by serotonin, although this drug has no apparent effects on basal neurotransmitter release [107]. The hypomorphic *Ih* mutant reduces the amplitude of the evoked response at the neuromuscular junction (NMJ) of third instar larvae by decreasing the number of released vesicles but exerted no apparent effects on NMJ morphology [107]. Therefore, the presynaptic HCN channel is active under basal conditions and increases neurotransmission at motor neuron terminals of larvae. The adult hypomorphic *Ih* mutant exhibits impaired locomotion, suggesting that the presynaptic HCN channel at NMJ functions in coordinated movement. The *Drosophila Ih* gene is orthologous to several human genes, including the *HCN1* to *4* genes. Of note, the *HCN1* gene is associated with EIEE (Table 1) [108]. Further analyses with *Drosophila* models targeting *Ih* may help to understand *HCN1*-associated EIEE.

#### 5.1.4. *Drosophila* Models Targeting Voltage-Gated Calcium Channels (Cav)

Voltage-gated calcium channels mediate the entry of calcium ions into excitable cells. They are also involved in many calcium-dependent processes such as muscle contraction, neurotransmitter release, gene expression, cell division, cell motility and cell death. The *Drosophila cacophony* (*cac*) gene encodes the α1 subunit of a voltage-gated calcium channel that is mainly localized at presynaptic active zones and functions in evoked neurotransmitter release at NMJ. The *cac* gene is responsible for male courtship behavior and numerous neurophysiological processes. Notably, a conditional *cac* mutant *cac^TS2^* exhibits rapid paralysis at elevated temperatures, having defects in neurotransmitter release [109]. *cac* has high homology with the human *calcium voltage-gated channel subunit α1A* (*CACNA1A*)*, CACNA1B and CACNA1E* genes. Among them, the *CACNA1A* and *CACNA1E* genes are associated with EIEE (Table 1) [110,111]. The *CACNA1B* gene is associated with neurodevelopmental disorder with seizures and nonepileptic hyperkinetic movements [112]. Based on the phenotypes observed with *cac^TS2^*, *Drosophila* models targeting *cac* may be suitable to examine *CACNA1A*- and *CACNA1E*-associated EIEE.

#### 5.1.5. *Drosophila* Models Targeting GABA_A_ Receptors

GABA is a well-known inhibitory neurotransmitter in the human brain and GABA_A_ receptors are heteropentameric, ligand-gated anion channels. GABA_A_ receptors are activated by GABA to mediate both phasic synaptic transmission and tonic extra-synaptic inhibition in the brain. GABA is also the major inhibitory neurotransmitter in the *Drosophila* nervous system. The *Drosophila* gene *CG8916* is an orthologue to several human GABA_A_ receptor genes, including *gamma-aminobutyric acid type A receptor subunit α1* (*GABRA1*), *GABRA2, GABRA4, GABRA5, GABRA6 and gamma-aminobutyric acid type A receptor subunit γ2* (*GABRG2*) genes. Among them, *GABRA1, GABRA2, GABRA5 and GABRG2* are associated with EIEE (Table 1). The *Drosophila Lcch3* gene is another orthologue to several human GABA_A_ receptor genes, including *gamma-aminobutyric acid type A receptor subunit β1* (*GABRB1*), *GABRB2 and GARB3*. Among them, *GABRB1* and *GABRB3* are associated with EIEE (Table 1). Although both the *CG8916* and *Lcch3* genes are predicted to be involved in several biological processes, including chemical synaptic transmission, chloride transmembrane transport and regulation of membrane potential, no phenotypic analysis with knockdown or mutant flies has been performed in relation to epilepsy [113]. Development of appropriate *Drosophila* models to investigate the roles of GABA_A_ receptors is required.

#### 5.1.6. *Drosophila* Models Targeting GABA_B_ Receptors

The *Drosophila GABA-B-R2* gene encodes metabotropic GABA_B_ receptor subtype 2 that exhibits G protein-coupled GABA receptor and protein heterodimerization activities. It is involved in several biological processes, such as the G protein-coupled receptor signal transduction pathway, cellular response to mechanical stimulus and negative regulation of secretion. GABA-B-R1/2 signaling has been characterized in astrocytes during *Drosophila* synaptogenesis [114]. Uptake of GABA by astrocytes mediated by GABA transporters is an essential mechanism in regulation of the excitatory/inhibitory balance in CNS. At the mid-pupal stage, CNS neuropil lacks astrocyte membranes and synapses. During synaptogenesis, astrocyte membranes infiltrate the neuropil. After forming synapses, GAT is upregulated in astrocytes. Disruption of *GABA-B-R1*/*2* signaling in astrocytes reduces the levels of GABA transporters in astrocytes. Notably, depletion of astrocytic GABA-B-R1/2 signaling in mutants suppressed mechanosensory-induced seizure activity with hyperexcitable neurons, suggesting that astrocytes actively alter the expression of GABA transporters via GABA-B-R1/2 signaling [114]. These studies suggest that the precise regulation of astrocytic GABA transporters and GABA-B-R1/2 signaling plays an important role in seizure activity. The human orthologue of the *Drosophila GABA-B-R2* gene is the *gamma-aminobutyric acid type B receptor subunit 2* (*GABBR2*), which is associated with EIEE (Table 1). The *Drosophila* models targeting *GABA-B-R2* may therefore be useful to gain more insight to *GABBR2*-associated EIEE.

#### 5.1.7. *Drosophila* Models Targeting NMDA Receptors

NMDA receptors (NMDR) are a subtype of ionotropic glutamate receptors. The *Drosophila* genome encodes two NMDAR homologues, NMDA receptor 1 (Nmdar1) and NMDA receptor 2 (Nmdar2), which form functional NMDAR similar to vertebrate NMDAR, including voltage-dependent activation by glutamate. *Nmdar1* is an orthologue of human *glutamate ionotropic receptor NMDA type subunit 1* (*GRIN1*) that is associated with autosomal dominant neurodevelopmental disorder with or without hyperkinetic movements and seizures. *Nmdar2* is orthologous to the human *glutamate ionotropic receptor NMDA type subunit 2B* (*GRIN2B*), *GRIN2C and GRIN2D* genes. Among them, *GRIN2B* and *GRIN2D* are associated with EIEE (Table 1). Although *Drosophila* NMDAR is involved in biological pathways, including memory, sleep and sensory perception of touch [115,116], no phenotypic analysis with knockdown or mutant flies has been performed in relation to epilepsy.

#### 5.1.8. *Drosophila* Models Targeting Solute Carrier Family

The *Drosophila Glucose transporter 1* (*Glut1*) gene encodes a transmembrane protein that transports glucose but not galactose and is involved in glucose homeostasis and positive regulation of peptide hormone secretion (https://flybase.org/reports/FBgn0264574). It is orthologous to several human genes, including *SLC2A1*, *SLC2A2, SLC2A3 and SLC2A4.* As described above, *SLC2A1* is associated with GLUT-1DS, although no phenotypic analysis with knockdown or mutant flies has been performed in relation to epilepsy.

The *Excitatory amino acid transporter 2* (*Eaat2*) gene encodes a transmembrane protein involved in aspartate and taurine transport. The human orthologue of this gene is *SLC1A2,* which is associated with EIEE (Table 1). Although it was reported that *Eaat2* is involved in biological processes, including chemosensitive behavior and sleep [117], no phenotypic analysis has been performed in relation to epilepsy.

Potassium/chloride cotransporters play cell-type specific roles in the regulation of several biological processes such as cell volume homeostasis, cell migration, neural circuit development and neuronal excitability. In humans, nine potassium/chloride cotransporters comprise the SLC12 family, SLC12A1–A9. In the *Drosophila* genome, there are five cotransporter genes—*kazachoc* (*kcc*), *ncc69*, *CG12773, CG31547 and CG10413*. The *kcc* gene is orthologous to human *SLC12A4*, *SLC12A5, SLC12A6 and SLC12A7*. Among them, *SLC12A5* is associated with EIEE (Table 1). The hypomorphic *kcc^DHS1^* mutant was identified as a seizure-enhancer of the bang-sensitive paralytic phenotypes of several seizure-sensitive mutants, including *para^bss1^* and *easily shocked* (*eas*) [118]. The young *kcc^DHS1^* flies also exhibit seizure-like behavior with a reduced threshold for seizures induced by electroconvulsive shock. Specific expression of wild-type *kcc* in the mushroom bodies (MBs) effectively suppressed the seizure-like phenotype of the *kcc^DHS1^* flies [119]. MBs play a central role in integrating incoming signals, such as olfactory, mechanical, taste and visual sensory signals and in sorting the distribution of outgoing motor signals [120]. *kcc^DHS1^* seizure sensitivity in MB neurons acts by weakening the chemical synaptic inhibition by GABAergic transmission, suggesting that this is due to disruption of intracellular Cl^―^ gradients in MB neurons. Glial cells were proposed to play important roles in the seizure disorders by maintaining extracellular ionic homeostasis in the nervous system [2]. Other group reported that glia- or neuron-specific knockdown of *kcc* reduced the threshold of seizure induction, accompanied by the induction of cell swelling and increase in brain volume in young adult flies [121]. Peripheral nerves of third instar larvae were also enlarged by neuron- or glia-specific knockdown of *kcc*. These observations suggest that a threshold of potassium/chloride cotransport dysfunction in the nervous system during development is an important determinant of seizure-susceptibility in *Drosophila*. The *kcc* mutants thus provide an excellent model system to elucidate the role of kcc in epileptogenesis related to GABAergic signaling and in glia.

SLC13 transmembrane transporters function in the transportation of di- and tricarboxylic acid and Krebs cycle intermediates such as succinate, citrate and alpha-ketoglutarate. The *Drosophila I’m not dead yet* (*Indy*) is orthologous to the human genes *SLC13A1*, *SLC13A2, SLC13A3, SLC13A4 and SLC13A5*. Among them, *SLC13A5* is associated with EIEE (Table 1). Mutations in *Indy* create a favorable metabolic state, similar to calorie restriction and extend the life span (https://flybase.org/reports/FBgn0036816). No phenotypic analysis of mutants and knockdown flies has been performed in relation to epilepsy.

SLC35 members are responsible for the transport of nucleotides and adenosine 3′-phospho 5′-phosphosulfate from the cytosol to the endoplasmic reticulum (ER) and the Golgi apparatus. These sugars are well-known substrates for the glycosylation and sulfation of proteins, lipids and proteoglycans. The *Drosophila UDP-galactose transporter* (*Ugalt*) gene encodes a transporter involved in UDP-N-acetylgalactosamine, UDP-galactose and galactose transmembrane transport (https://flybase.org/reports/FBgn0024994). It is orthologous to the human *SLC35A2* gene that is associated with EIEE (Table 1). No phenotypic analysis for *Ugalt* has been performed in relation to epilepsy.

*Drosophila Glutamate Carrier 1* (*GC1*) gene encodes a carrier protein of the inner mitochondrial membrane that transports L-glutamate across the inner mitochondrial membrane [122]. It is orthologous to the human *SLC25A18* and S*LC25A22* genes. Out of them, the S*LC25A22* gene is associated with EIEE (Table 1). No phenotypic analysis with knockdown or mutant flies has been performed in relation to epilepsy.

*Drosophila aralar1* gene encodes a solute carrier protein playing a role as a carrier exchanging a glutamate and a proton (H^+^) from the cytoplasm for an aspartate in mitochondria. Recently, it is reported that Aralar1 is responsible for uptake of GABA into mitochondria that is activated by mitochondrial membrane polarization. Interestingly, heterozygous *aralar1* mutant flies exhibit decreased competition for food and grooming and hyperactivity, indicating a role in social activity [123]. The *aralar1* gene is orthologous to the human *SLC25A12* and S*LC25A13* genes. Out of them, the S*LC25A12* gene is associated with EIEE (Table 1). The *Drosophila* models targeting *aralar1* would be useful for further investigation of a role of *SLC25A12* in pathogenesis of EIEE.

#### 5.1.9. *Drosophila* Models Targeting Synaptic Vesicle Release/Membrane Trafficking

Extensive behavioral screens to isolate mutations of genes essential for synaptic transmission in *Drosophila* have identified temperature-sensitive mutations causing paralysis at a restrictive temperature [79,124]. One such mutant was named as *shibire* (*shi*). Later, the *shibire* gene was found to be a *Drosophila* orthologue of human *dynamin 1* (*DNM1*) and *dynamin 2* (*DNM2*) (Table 1). Dynamin 1 is a GTPase involved in clathrin-mediated endocytosis of synaptic vesicles [125]. De novo mutations in the *DNM1* gene have been found in patients with DEE, including LGS and WS [5,126] and *DNM1* is associated with EIEE (Table 1). The G domain structure of DNM1 can be destabilized by these mutations, resulting in impaired nucleotide binding, dimer formation, and/or GTPase activity of DNM1 [126]. The mutant carries a temperature-sensitive missense mutation in the GTPase domain of the protein. When *shi^ts1^* mutants are heated to the restrictive temperature and stimulated, their nerve terminals become depleted of synaptic vesicles and flies become paralyzed [125,127]. In the *shi^ts1^* mutant flies, the presynaptic membrane at nerve terminals is filled with clathrin-coated pits, suggesting that vesicles are stuck at the late stage of endocytosis [128]. When flies are placed at a permissive temperature again, dynamin resumes vesicular recycling. In addition to defects in vesicle endocytosis, it was also reported that ionic currents recorded from neuromuscular synapses of the dorsal longitudinal muscle in *shi^ts1^* mutant flies exhibit rapid synaptic fatigue to high-frequency stimulation. These observations with *Drosophila* models suggest an additional role for dynamin in maintenance of the releasable pool of vesicles [129].

The *Drosophila Ras opposite* (*Rop*) belongs to the STXBP/unc-18/SEC1 family that interacts with SNAREs to regulate assembly of the SNARE complex containing synaptobrevin, syntaxin and SNAP-25. The SNARE complex mediates neurotransmitter release by responding to presynaptic entry of Ca^2+^. Rop is also responsible for regulation of synaptic vesicle exocytosis. *Rop* is orthologous to human *STXBP1*, *STXBP2 and STXBP3* genes. The *STXBP1* gene is associated with EIEE (Table 1). Electrophysiological analyses revealed that temperature-sensitive *Rop* mutants have a loss of the synaptic response to a light stimulus at a restrictive temperature [130]. Of note, *C. elegans unc-18* mutants exhibit a paralytic phenotype [131]. Studies with transgenic fly lines overexpressing Rop and syntaxin revealed that Rop interacts with syntaxin in vivo *and* is a rate-limiting factor of exocytosis, playing both positive and inhibitory roles in neurotransmission [132]. In addition, a role of Rop in dendrite growth was reported. During neuronal development, axons and dendrites become longer by several orders of magnitude. In loss-of-function *Rop* mutants, neurons have reduced terminal dendrite outgrowth followed by primary dendrite degeneration, suggesting that Rop plays a key role during dendrite development [133]. In addition, mis-localization of syntaxin-1 and reduced neurite extension were observed in an EIEE patient-derived iPSC model [134]. *Drosophila* models targeting *Rop* thus provide a useful tool for studying *STXBP1*-associated EIEE.

The *Drosophila complexin* (*cpx*) gene encodes a presynaptic cytosolic protein that regulates the assembly and function of the SNARE complex. Analyses of *cpx* mutants revealed that cpx acts both positively and negatively in synaptic transmission, serving as a clamp of the synaptic vesicle fusion and also as an activator of evoked release [135,136]. Cpx is phosphorylated by protein kinase A (PKA) and controls activity-dependent spontaneous neurotransmitter release and plasticity of synapse structure [137]. *cpx* is orthologous to the human *complexin 1* (*CPLX1*) and *CPLX2* genes. *CPLX1* is associated with EIEE (Table 1). Further analyses with *Drosophila* models targeting *cpx* in relation to *CPLX1*-associated EIEE may be of interest.

The *Drosophila CG9132* gene is involved in vesicle-mediated transport and predicted to localize to the clathrin vesicle coat (https://flybase.org/reports/FBgn0030791). Human orthologues of this gene are *NECAP endocytosis associated 1* (*NECAP1*) and *NECAP2* genes. The *NECAP1* gene is associated with EIEE (Table 1). No phenotypic data of mutants or knockdown flies for *CG9132* has been reported in relation to epilepsy.

The *Drosophila milton* (*milt*) gene encodes a scaffolding protein forming a protein complex with Miro that links kinesin heavy chain to mitochondria to regulate axonal transport of mitochondria [138,139]. Human orthologues of this gene are *trafficking kinesin protein 1* (*TRAK1*) and *TRAK2*. The *TRK1* gene is associated with EIEE (Table 1). No phenotypic data of mutants or knockdown flies for *milt* has been reported in relation to epilepsy.

Adaptor protein (AP) complexes select cargo for inclusion into coated vesicles at the late stage of secretory and endocytic pathways. The adaptor protein-3 (AP-3) complex is mainly involved in the biogenesis of lysosome-related organelles. The *Drosophila ruby* (*rb*) gene encodes the β3-adaptin subunit of the AP-3 complex with cargo adaptor activity. The *rb* gene is required for regulation of lipid storage, eye pigment biogenesis and processing of the Notch receptor [140]. The *rb* gene is orthologous to human *adaptor related protein complex 3 subunit β1* (*AP3B1*) and *AP3B2*. *AP3B1* is associated with Hermansky-Pudlak Syndrome, which is characterized by platelet defects and oculocutaneous albinism. *AP3B2* is associated with EIEE (Table 1).

*Drosophila* synaptojanin (Synj) is a lipid phosphatase responsible for synaptic vesicle trafficking. The protein has two distinct lipid phosphatase domains called the 5-phosphatase domain and SAC1 domain, each of them targets different phosphoinositide phosphate (PtdInsP) species. The 5-phosphatase domain of Synj specifically hydrolyzes phosphates at the 5′ position of the inositol ring and thus exhibits a preference for PI(4,5)P2 as a substrate. In contrast, the SAC1 domain of Synj hydrolyzes PI(3)P, PI(4)P and PI(3,5)P2. Synj is involved in several biological processes, including the maintenance of presynaptic active zone structure, positive regulation of autophagosome assembly and synaptic vesicle endocytosis. Its human orthologues are synaptojanin 1 (SYNJ1) and SYNJ2. Mutations in the human *SYNJ1* gene are associated with both Parkinson’s disease (PD) and EIEE (Table 1). *Drosophila* PD models targeting *Synj* have been developed to demonstrate its role in autophagosome maturation at presynaptic terminals, linking presynaptic-specific autophagy defects to PD [141]. Development of fly EIEE models targeting *Synj* is necessary to shed light on a possible role of *SYNJ1* in EIEE pathogenesis.

*Drosophila skywalker* (*sky*) encodes a RabGAP that activates GTPase activity of Rab35. Sky restricts the ability of synaptic vesicles to fuse into a synaptic endosomal compartment, thereby limiting synaptic vesicle-associated protein sorting at synaptic endosomes. In this process, the binding of sky to phosphoinositides and membrane-association is required. *sky* is orthologous to the human *TBC1 domain family member 24* (*TBC1D24*) gene that is associated with EIEE (Table 1). In sky mutants, synaptic vesicles travel excessively to endosomes to cause defects in neurotransmission and neurodegeneration. Reduction of endosome-to-lysosome trafficking controlled by the homotypic fusion and vacuole protein sorting (HOPS) complex rescued these defects in the *sky* mutants [142]. The synaptic transmission is thus promoted by effective turnover of protein at lysosomes, suggesting a novel target to suppress defects caused by mutations in the human *TBC1D24* gene. Other studies with *Drosophila* models revealed that the most common mutations in the patients affected the phosphoinositide-binding pocket of sky to inhibit its lipid binding in the membrane, resulting in diffusion of sky into presynaptic terminals [143]. Moreover, these pathogenic mutations induced severe neurological defects in *Drosophila* such as defective synaptic-vesicle trafficking and seizures. These defects were effectively suppressed by *SynJ* mutations that increased PI(4,5)P2 concentrations in the synapse. Thus, these *Drosophila* models targeting *sky* may be useful to further explore the pathogenic mechanisms of *TBC1D24*-associated EIEE.

#### 5.1.10. *Drosophila* Models Targeting Cell Adhesion Molecules

Disintegrin and metalloprotease (ADAM) proteins are a metzincin superfamily of metalloproteases. ADAM proteins contain conserved domains such as a prodomain, metalloprotease domain, disintegrin-like domain, cysteine rich domain, EGF-like domain, transmembrane domain and a cytoplasmic domain. The metalloprotease domain is responsible for ectodomain shedding to release the ectodomains of many membrane proteins. *Drosophila* mind-meld (mmd) is predicted to have such metalloendopeptidase activity to be involved in the ectodomain shedding of membrane proteins (https://flybase.org/reports/FBgn0259110). Its human orthologues are *ADAM11, ADAM22 and ADAM23*. Among them, *ADAM22* is associated with EIEE (Table 1). No phenotypic analysis of *mmd* mutants or knockdown flies has been reported in relation to epilepsy.

#### 5.1.11. *Drosophila* Models Targeting Cytoskeletal Protein

The *Drosophila α-Spectrin* (*α-Spec*) gene encodes an essential protein that interacts with the products of *β-Spectrin* (*β-Spec*) or *karst* (*kst*) to form a cytoskeletal complex that is associated with the plasma membrane. It is involved in a lipoprotein pathway and asymmetric division of germ line stem cells via spectrosomes and fusomes. It is also involved in long-term strengthening of neuromuscular junction and nervous system development. The postsynaptic knockdown of α- or β-Spectrin increased the size of the active zone and perturbed its spacing, suggesting that a postsynaptic Spectrin–actin lattice acts as an organizing scaffold to control pre- and post-synaptic development [144]. Human orthologues of this gene are the *SPTAN1* and the *spectrin alpha, erythrocytic 1* (*SPTA1*) genes. As described above, the *SPTAN1* gene is associated with EIEE (Table 1). Studies with a *Drosophila* α-synucleinopathy (Parkinson’s disease) model revealed that α-synuclein interacts with Spectrins to destabilize the actin cytoskeleton and induce mitochondrial dysfunction [145]. Although no phenotypic analysis of *α-Spectrin* mutants has been reported in relation to epilepsy, *Drosophila* models targeting *α-Spectrin* may be useful to examine *SPTAN1*-associated EIEE.

*Drosophila CG32264* encodes a protein predicted to localize to the cytosol and nucleus. It is also predicted to function in actin cytoskeleton reorganization (https://flybase.org/reports/FBgn0052264). It is orthologous to human *phosphatase and actin regulator 1* (*PHACTR1*) and *PHACTR2* genes. The *PHACTR1* gene is associated with EIEE (Table 1). No phenotypic data of mutants or knockdown flies for *CG32264* has been reported.

#### 5.1.12. *Drosophila* Models Targeting Intracellular Signal Transduction

Guanine nucleotide-binding proteins (G proteins) act as regulators or transducers in a variety of transmembrane signaling pathways. The *Drosophila G protein α o subunit* (*Gαo*) gene encodes the G protein that is most abundant in nervous tissues, exhibiting GTP binding activity. It is orthologous to the human *G protein subunit alpha o1* (*GNAO1*) gene that is associated with EIEE (Table 1). Gαo plays a role in a variety of biological processes such as behavioral response to starvation, negative regulation of synaptic growth at NMJ and development of the nervous system. Of note, it also plays a role in the differentiation of glial cells and formation of the blood brain barrier in *Drosophila* [146]. The blood-brain barrier of *Drosophila* is formed by the surface glia, ensheathing the nerve cord and insulating it against the potassium-rich hemolymph by establishing intercellular septate junctions. If the barrier does not work properly, action potentials cannot propagate and the fly is paralyzed. Gαo and another G protein subunit Gαi, the G protein-coupled receptor (GPCR) Moody and Loco, the G protein signaling regulator, are all required in surface glia for effective insulation. Although further analysis is necessary, these observations with *Drosophila* models may provide a possible mechanism to understand the pathogenesis of the *GNAO1*-assaociated EIEE.

Rho (Ras Homologous) GTPases are the Ras superfamily members of small GTPases. It is well known that typical Rho GTPases play a role in synaptic plasticity and cognitive function. However, a possible role of atypical Rho GTPases, such as Rho-related BTB domain containing 1 (RHOBTB1) and RHOBTB2, in neurodevelopment has not been elucidated. *Drosophila* RhoBTB, which was suggested to be involved in the anti-parasitoid immune response [147], is an orthologue of the human RHOBTB1 and RHOBTB2. The *RHOBTB2* gene is associated with EIEE (Table 1). When expressed in HEK293 cells, the level of RHOBTB2 carrying mutations found in the EIEE patients was higher than that of the wild-type protein. *Drosophila* models have been developed by establishing transgenic flies overexpressing RhoBTB by the GAL4-UAS targeted expression system [148]. The transgenic flies overexpressing RhoBTB in pan-neurons demonstrated high bang sensitivity and were paralyzed after vortex, indicating the seizure-susceptible phenotype [148]. Overexpression of RhoBTB in pan-neurons, motoneurons, muscle and glia all induced severe locomotive defects [148]. In addition, dendritic arborization neuron-specific knockdown of RhoBTB induced a decreased number of dendrites, suggesting a role of RhoBTB in the development of dendritic neurons. These *Drosophila* models may be useful to study *RHOBTB2*-associated EIEE.

*Drosophila Phospholipase C at 21C* (*Plc21C*) encodes a phospholipase C β that functions downstream of G-protein-coupled receptors involved in a variety of biological pathways such as olfactory transduction, entrainment of circadian clock by photoperiod, flight behavior and regulation of synaptic transmission at NMJ [149]. *Plc21C* is orthologous to human *phospholipase C β1* (*PLCB1*) and *PLCB3* genes. *PLCB1* is associated with EIEE (Table 1). No phenotypic analysis of *Plc21C* mutants or knockdown flies has been reported in relation to EIEE.

RhoGEFs are guanine nucleotide exchange factors (GEFs) for Rho family GTPases. *Drosophila zizimin-related* (*Zir*) encodes a Rho guanine nucleotide exchange factor involved in the innate immune response [150]. Its human orthologues are *dedicator of cytokinesis 6* (*DOCK6*), *DOCK7 and DOCK8* genes. The *DOCK7* gene is associated with EIEE (Table 1). No phenotypic analysis of *Zir* mutants or knockdown flies has been reported in relation to EIEE.

The protein encoded by *Drosophila pinstripe* (*pns*) is predicted to be GEF for the Rab family GTPase (https://flybase.org/reports/FBgn0035229). It is also predicted to localize to the Golgi apparatus and function in the regulation of Rab signal transduction. Its human orthologues are *DENN domain containing 5A* (*DENND5A*), *DENND5B* and *DENND4C* genes. Among them, DENND5A is associated with EIEE (Table 1). No phenotypic analysis of *pns* mutants or knockdown flies has been reported in relation to EIEE.

The 14-3-3 proteins are highly conserved acidic 30-kDa homo/heterodimeric adapter proteins that bind and regulate protein activities. *Drosophila* 14-3-3ε encodes a protein that exhibits phosphoserine residue binding activity, protein heterodimerization activity and binding activity to many transcription factors, regulating a number of signaling pathways, including the Ras/MAPK pathway. It is involved in embryonic hatching, germ cell migration, gonad formation, wing venation and eye development. It is orthologous to human *tyrosine 3-monooxygenase/tryptophan 5-monooxygenase activation protein epsilon* (*YWHAE*), which is associated with EIEE (Table 1). A role of 14-3-3ε in axon guidance has been reported [151]. Among the families of axon guidance cues, Semaphorins play roles in sculpting the nervous system by serving as axonal repellents. Semaphorins utilize Plexin receptors to exert repulsive effects on the extension of axons. Plexins are Ras/Rap family GTPase activating proteins (GAPs). The GAP domain of Plexin A is phosphorylated by PKA. This Plexin A phosphorylation generates a specific binding site for 14-3-3ε. This complex formation inhibits interaction of Plexin A with its substrate and antagonizes the repulsive effects of Semaphorin on axon extension. A role of 14-3-3ε in polyglutamine diseases was also reported with *Drosophila* models [152]. Spinocerebellar ataxia type 1 (SCA1) and HD are polyglutamine disorders caused by expansion of a CAG repeat within the coding regions of the Ataxin-1 and Huntingtin proteins, respectively. Transgenic flies expressing human Ataxin-1 protein containing a polyglutamine tract of 82 glutamines in eye imaginal discs induced abnormal eye morphology (rough eye phenotype). Similarly, flies overexpressing the N-terminal portion of the human Huntingtin protein (amino acids 1-336) including an expanded tract of 128 glutamine repeats induced the rough eye phenotype. Co-expression of *Drosophila 14-3-3ε* enhanced the rough eye phenotype, whereas loss-of-function mutation of *14-3-3ε* suppressed the rough eye phenotype. Considering these uses of the *14-3-3ε* transgenic lines and mutants in the study of polyglutamine diseases, utilization of these flies in the study of the *YWHAE*-associated EIEE is expected.

*Drosophila specifically Rac1-associated protein 1* (*Sra-1*) encodes an essential protein that is a component of the WAVE regulatory complex. The WAVE regulatory complex controls dynamics of actin cytoskeleton by promoting the actin-nucleating activity of the Arp2/3 complex at distinct membrane sites. Sra-1 interacts with the Fragile X mental retardation protein (FMRP) encoded by the *Fmr1* gene, the translation initiation factor eIF4E and Rho GTPase Rac1 to control morphogenesis and synapse organization. Similar to mutations in *Fmr1* and *Rac1,* mutations in *Sra-1* also affect axons and synapses [153]. Sra-1 acts as a Rac1 effector that antagonizes FMRP function, revealing a link between signal-dependent remodeling of the cytoskeleton and translation [154,155]. *Drosophila Sra-1* is orthologous to the human *cytoplasmic FMR1 interacting protein 1* (*CYFIP1*) and *CYFIP2* genes. The *CYFIP2* gene is associated with EIEE (Table 1). No phenotypic analysis of *Sra-1* mutants or knockdown flies has been reported in relation to EIEE.

*Drosophila rabconnectin-3A* (*Rbcn-3A*) encodes a protein that is part of a regulatory subunit of the vacuolar H^+^ ATPase required for acidification of intracellular vesicles and the lysosome. Rbcn-3A is involved in endosomal trafficking and lysosome function via regulation of vacuolar H^+^ ATPase function [156]. Genetic screening based on the number and morphology of peroxisomes in *Drosophila* identified the *Rbcn-3A* mutations that increased the number of peroxisomes [157]. Peroxisomes are the sites of a variety of oxidative reactions and their dysfunction affects the nervous system. Mutations in *Rbcn-3A* also inhibit Notch signaling in follicle cells and imaginal disc cells. *Rbcn-3A* is orthologous to human *Dmx like 1* (*DMXL1*) and *DMXL2* genes. The *DMXL2* gene is associated with EIEE (Table 1). It is not known yet which *Rbcn-3A*-related pathway is responsible for the pathogenesis of EIEE. Further analyses with *Drosophila* models targeting *Rbcn-3A* are required to clarify this point.

#### 5.1.13. *Drosophila* Models Targeting Transcription Factors

*Drosophila aristaless* (*al*) gene encodes a paired-like (PRD-like) homeobox transcription factor with sequence-specific DNA binding activity to regulate transcription [158,159]. It is involved in chaeta development and imaginal disc morphogenesis. It is orthologous to the human *ARX* gene that is associated with a spectrum of disorders, from EIEE to non-syndromic mental retardation, as described above (Table 1). No phenotypic analysis of *Drosophila al* mutants or knockdown flies has been reported in relation to EIEE.

*Drosophila cut* (*ct*) encodes a homeodomain protein that is a transcription factor functioning in numerous tissues such as wing discs, muscles, oocytes and neurons. It is a regulator of type-specific neuronal identity in the peripheral nervous system. *Ct* is expressed at variable levels in the dendritic arborization neurons and these levels control the different dendritic morphologies through regulation of the cytoskeleton [160,161]. It is orthologous to human *cut like homeobox 1* (*CUX1*) and *CUX2* genes. *CUX2* is associated with EIEE (Table 1). Further studies with *Drosophila* models targeting *ct* may be necessary to clarify the link between *CUX2* and EIEE.

#### 5.1.14. *Drosophila* Models Targeting Translation

*Drosophila eukaryotic translation elongation factor 1 α 2* (*eEF1α2*) encodes a protein that is predicted to have GTPase activity and translation elongation factor activity (https://flybase.org/reports/FBgn0000557). The human orthologue of *Drosophila eEF1α2* gene is human *eukaryotic translation elongation factor 1 α 2* (*EEF1A2*), which is associated with autosomal dominant non-syndromic intellectual disability and EIEE (Table 1). No phenotypic analysis of *eEF1α2* mutants or knockdown flies has been reported.

Mitochondrial aminoacyl-tRNA synthetases catalyze the ligation of amino acids to their cognate tRNAs in mitochondria. They are encoded by nuclear genes and are imported into mitochondria with the guidance of a mitochondrial targeting sequence. *Drosophila alanyl-tRNA synthetase* (*AlaRS*) encodes a protein that is predicted to be involved in alanyl-tRNA aminoacylation (https://flybase.org/reports/FBgn0027094). *Drosophila prolyl-tRNA synthetase, mitochondrial* (*ProRS-m*) encodes a protein that is predicted to be involved in prolyl-tRNA aminoacylation (https://flybase.org/reports/FBgn0027082). Both proteins are also predicted to localize to mitochondria. *AlaRS* is orthologous to human *alanyl-tRNA synthetase 1* (*AARS1*), which is implicated in Charcot-Marie-Tooth disease and EIEE (Table 1). *ProRS-m* is orthologous to human *prolyl-tRNA synthetase 2, mitochondrial* (*PARS2*), which is associated with EIEE (Table 1). Although no phenotypic analysis of mutants or knockdown flies for *AlaRS* and *ProRS-m* has been reported, several defects in mitochondrial function may be related to EIEE.

*Drosophila waclaw* (*waw*) is predicted to have GTP binding activity and GTPase activity. Its human orthologue is the *GUF1* gene encoding a mitochondrial elongation factor to regulate the fidelity of translation by catalyzing a one-codon backward translocation of tRNAs on improperly translocated ribosomes [162]. The *GUF1* gene is associated with EIEE (Table 1). No phenotypic data on *waw* mutants and knockdown flies are available.

#### 5.1.15. *Drosophila* Models Targeting Post-Translational Modification

*Drosophila ubiquitin-like activating enzyme 5* (*Uba5*) encodes a member of the E1-like ubiquitin-activating enzyme family. It activates the ubiquitin-fold modifier 1 (UFM1) that forms a thioester bond with an E2 cofactor, UFC1, that results in the tagging of reactive ubiquityl units to substrates by action of an E3 ligase, UFL1. The *Drosophila Uba5* gene is orthologous to the human *ubiquitin like modifier activating enzyme 5* (*UBA5*) gene. Human *UBA5* is associated with autosomal recessive spinocerebellar ataxia-24 (SCAR24) and EIEE (Table 1). *Drosophila* models targeting *Uba5* have been developed [163]. Neuron-specific knockdown of *Uba5* induced a reduction of locomotor activity evaluated by climbing assay and flight assay, a shortened lifespan and morphological defects in NMJ such as a reduced number of type Ib boutons and increased size of boutons. Both *Ufm1* and *Ufc1* knockdown flies also exhibit similar phenotypes. Of note, both *Drosophila* wild-type *Uba5* and human wild-type *UBA5* but not mutated genes, rescued the defects at NMJ. Although the transgenic flies have been characterized as a model for SCAR24, further analyses, such as Bang-sensitivity assay and learning assay, may be of interest for evaluation as a *Uba5*-associated EIEE model.

The *Drosophila godzilla E3 ubiquitin protein ligase* (*gzl*) gene encodes a member of the RNF family of membrane-anchored E3 ubiquitin ligases that ubiquitylates synaptobrevin, a component of the SNARE complex mediating synaptic vesicle release [164]. It also promotes apico-basal transcytosis of the product of *wingless* in the wing imaginal disc [165]. Human orthologues of *gzl* are the *ring finger protein 13* (*RNF13*) and *RNF167* genes. The *RNF13* gene is associated with EIEE (Table 1). Although no phenotypic analysis of *gzl* mutants or knockdown flies has been reported in relation to EIEE, defects in synaptic vesicle exocytosis are expected in the link between *RNF13* and EIEE.

*Drosophila Alg13 UDP-N-acetylglucosaminyltransferase subunit* (*Alg13*) encodes a protein that is predicted to be involved in dolichol-linked oligosaccharide biosynthetic process (https://flybase.org/reports/FBgn0039639). Dolichyl phosphate N-acetylglucosaminyltransferases add a second N-acetylglucosamine (GlcNAc) residue to the dolichol pyrophosphate (PP-Dol) lipid carrier during the process of protein N-glycosylation. This activity is carried out by the Alg13/Alg14 complex. The human orthologue of *Drosophila Alg13* is the human *ALG13 UDP-N-acetylglucosaminyltransferase subunit* (*ALG13*) gene, which is implicated in EIEE (Table 1). No phenotypic analysis of *Drosophila Alg13* mutants or knockdown flies has been reported. In *Drosophila*, there is another gene called *ovarian tumor* (*out*) that is similar to the human *ALG13*. However, as *out* is slightly more similar to the human *OTU deubiquitinase 4* (*OTUD4*) gene, it may not be orthologous to the human *ALG13.* Further analysis is necessary to clarify this point.

*Drosophila PIG-Q*, *PIG-P*, *PIG-B and PIG-A* genes encode proteins required for GPI-anchor biosynthesis. Human orthologues of *Drosophila PIG-Q, PIG-P, PIG-B and PIG-A* are human *PIGQ*, *PIGP*, *PIGB and PIGA*, respectively. The human *PIGQ*, *PIGP*, *PIGB and PIGA* genes are associated with EIEE (Table 1). Although no phenotypic analysis of mutants or knockdown flies of *Drosophila PIG-Q, PIG-P or PIG-A* has been reported, they are predicted to localize to the ER. In contrast, *Drosophila* PIG-B localizes to the nuclear envelope (NE). Transgenic flies expressing the ER-localized form of PIG-B and NE-localized form of PIG-B have been established [166]. Expression of the ER-localized form of PIG-B inefficiently rescued the lethality of the *PIG-B* mutant, whereas the NE-localized form of PIG-B efficiently rescued this lethality, suggesting that the NE localization is essential for its function. The region of the ER proximal to the NE may be the translation site of many GPI-anchored proteins and GPI addition. It is therefore suggested that the NE and the proximal ER provide a platform for effective GPI anchoring. Further studies with *Drosophila* models are expected to address the link between the *PIG* genes and EIEE.

#### 5.1.16. *Drosophila* Models Targeting Epigenetic Factors

*Drosophila brahma associated protein 55 kD* (*Bap55*) encodes a member of two distinct chromatin remodeling complexes, the Brahma complex and Tat interactive protein 60 kD (TIP60) complex. In the Brahma complex, Bap55 is responsible for cell growth and survival in the wing imaginal disc. In the TIP60 complex, it is required to regulate dendrite wiring specificity in *Drosophila* olfactory projection neurons [167]. Bap55 is orthologous to human *actin like 6A* (*ACTL6A*) and *ACTL6B* genes. The *ACTL6B* gene is associated with EIEE (Table 1). Of note, post mitotic expression of *Bap55* or its human orthologues *ACTL6A* and *ACTL6B* effectively rescued the neuronal phenotype of the *Bap55* mutants. Further studies with *Drosophila* models targeting *Bap55* may be of interest to clarify the link between *ACTL6B* and EIEE.

#### 5.1.17. *Drosophila* Models Targeting Mitochondrial Enzymes

*Drosophila malate dehydrogenase 2* (*Mdh2*) encodes one of the enzymes in the tricarboxylic acid cycle in mitochondria (https://flybase.org/reports/FBgn0262559). It is orthologous to human malate dehydrogenase 2 (MDH2) that is associated with EIEE (Table 1). No phenotypic analysis of *Mdh2* mutants or knockdown flies has been reported in relation to EIEE.

*Drosophila Glutaminase* (*GLS*) is predicted to have glutaminase activity and localize to mitochondria (https://flybase.org/reports/FBgn0261625). It is involved in the glutamate biosynthetic process and glutamine catabolic process. It is orthologous to human *glutaminase* (*GLS*) and *GLS2* genes. The human *GLS* gene is associated with EIEE (Table 1). No phenotypic data of mutants or knockdown flies for *Drosophila GLS* has been reported.

The *Drosophila glutamate oxaloacetate transaminase 2* (*Got*) gene encodes an L- aspartate:2-oxoglutarate aminotransferase involved in glutamate biosynthesis, neurotransmitter receptor metabolic process and regulation of postsynaptic receptor field size [168]. It localizes to mitochondria. It is orthologous to the human *glutamic-oxaloacetic transaminase 2* (*GOT2*) gene, which is associated with EIEE (Table 1). No phenotypic data of mutants or knockdown flies for *Drosophila Got* has been reported in relation to EIEE.

#### 5.1.18. Others

The *Drosophila polynucleotide kinase 3’-phosphatase* (PNKP) (*CG9601*) gene encodes a protein predicted to have nucleobase-containing compound kinase activity and polynucleotide 3’-phosphatase activity. It may be involved in DNA repair and nucleotide phosphorylation (https://flybase.org/reports/FBgn0037578). The human orthologue of this gene is *polynucleotide kinase 3’-phosphatase* (*PNKP*), which is implicated in microcephaly, seizures and developmental delay and EIEE (Table 1). No phenotypic data of mutants or knockdown flies for *CG9601* has been reported.

*Drosophila rudimentary* (*r*) encodes the carbamoyl-phosphate synthetase 2, aspartate transcarbamylase and dihydroorotase (CAD) protein that catalyzes the initial steps of de novo pyrimidine biosynthesis. Mutations in the *r* gene induce wing malformations and pyrimidine auxotrophy [169]. The *Drosophila r* gene is orthologous to human *carbamoyl-phosphate synthetase 2, aspartate transcarbamylase and dihydroorotase* (*CAD*) genes that are associated with EIEE (Table 1). No phenotypic analysis of *r* mutants or knockdown flies has been reported in relation to EIEE.

The product of *Drosophila CG8891* is predicted to have nucleoside-triphosphate diphosphatase activity and to be involved in the nucleoside triphosphate catabolic process (https://flybase.org/reports/FBgn0031663). It is orthologous to the human *inosine triphosphatase* (*ITPA*) gene. ITPA may be responsible for excluding non-canonical purines from RNA and DNA precursor pools. The *IPTA* gene is associated with EIEE (Table 1). No phenotypic analysis of *CG8891* mutants or knockdown flies has been reported.

*Drosophila CG30122* encodes a protein predicted to localize to the precatalytic spliceosome and be involved in mRNA splicing. Human orthologues of this gene are *heterogeneous nuclear ribonucleoprotein U like 1* (*HNRNPUL1*), *HNRNPUL2 and heterogeneous nuclear ribonucleoprotein U* (*HNRNPU1*). Among them, the *HNRNPU1* gene is implicated in EIEE (Table 1). Genetic screening of RNAi lines that alter the TBPH-induced abnormal eye morphology phenotype identified the *CG30122* gene as a strong enhancer of the phenotype [170]. TBPH is a *Drosophila* orthologue of TDP-43, the hnRNP protein involved in amyotrophic lateral sclerosis (ALS) and frontotemporal dementia (FTD), suggesting the possible involvement of human *CG30122* orthologues in ALS/FTD. However, candidate target genes/mRNAs of *CG30122* hnRNP relating with EIEE are not known.

The *Drosophila WW domain containing oxidoreductase* (*Wwox*) gene encodes a protein that functions in homeostasis by controlling the balance between oxidative phosphorylation and glycolysis. It localizes to the cytoplasm and is involved in processes, including defense response to Gram-negative bacteria, control of reactive oxygen species metabolic process and response to ionizing radiation [171]. It is orthologous to the human *WW domain containing oxidoreductase* (*WWOX*) gene. The human *WWOX* gene is implicated in EIEE (Table 1). No phenotypic analysis of *Wwox* mutants or knockdown flies has been reported in relation to EIEE.

The *Drosophila ACAT-related protein required for viability 1* (*Arv1*) gene encodes a protein predicted to localize to the Golgi apparatus and cortical ER (https://flybase.org/reports/FBgn0052442). It is also predicted to be involved in biological processes, including the sphingolipid metabolic process, regulation of plasma membrane sterol distribution, sterol metabolic process, intracellular sterol transport and positive regulation of cell division. The *Drosophila Arv1* is orthologous to the human *ARV1 homolog fatty acid homeostasis modulator* (*ARV1*), which is associated with EIEE (Table 1). No phenotypic analysis of *Arv1* mutants or knockdown flies has been reported in relation to EIEE.

The *Drosophila canopy b* (*CNPYb*) gene encodes a protein that exhibits chaperone binding activity and is involved in chaperone-mediated protein folding. It is localized to the endomembrane system. Human orthologues of this gene are *canopy FGF signaling regulator 3* (*CNPY3*) and *CNPY4*. The *CNPY3* gene is associated with EIEE (Table 1). Mammalian ER-resident chaperone gp96 requires the cochaperone CNPY3 for proper folding and expression of Toll-like receptors (TLRs). Similarly, *Drosophila* gp93 requires *CNPYb* for proper folding and expression of TLR [172]. No phenotypic analysis of *CNPYb* mutants or knockdown flies has been reported in relation to EIEE.

The *Drosophila krueppel target at 95D* (*KrT95D*) gene encodes a protein that is predicted to be involved in protein localization to the plasma membrane (https://flybase.org/reports/FBgn0020647). Human orthologues of this gene are human *phosphofurin acidic cluster sorting protein 1* (*PACS1*) and *PACS2* genes. The *PACS1* gene is implicated in Schuurs-Hoeijmakers Syndrome and the *PACS2* gene is associated with EIEE (Table 1). No phenotypic data of mutants or knockdown flies for *KrT95D* has been reported.

*DrosophilaUDP-glucose pyrophosphorylase* (*UGP*) encodes an enzyme that synthesizes UDP-galactose from uridine triphosphate and galactose-1-phosphate. It is localized in the cytosol. *Drosophila UGP* mutants have been characterized in the development of *Drosophila* galactosemia models [173]. The *UGP* mutants exhibit defects in locomotion and morphology of NMJ such as expanded synaptic arbors accompanied with glycosylation losses and changes in the *Wnt* trans-synaptic signaling. It was also reported that the *Drosophila UGP* is involved in response to hyperoxia [174]. The *Drosophila UGP* gene is orthologous to the human *UDP-glucose pyrophosphorylase 2* (*UGP2*) gene, which is associated with EIEE (Table 1). Further studies with *Drosophila* models targeting *UGP* may be useful to clarify the link between *UGP2* and EIEE.

*Drosophila sugarless* (*sgl*) encodes UDP-glucose 6-dehydrogenase that is required for generating UDP-glucuronate, the substrate for glucuronate addition to glycosaminoglycan chains. Sgl is involved in several biological processes, including ameboidal-type cell migration, proteoglycan biosynthetic process and segment polarity determination. Phenotypic analysis of *sgl* revealed that *sgl* is important for the biosynthesis of both chondroitin sulfate and heparan sulfate and is likely the only gene providing UDP-glucose dehydrogenase activity in *Drosophila* [175]. The role of sgl in synaptogenesis was also reported in other studies [176]. Synaptogenesis depends on trans-synaptic signals that are regulated by the carbohydrate environment in the synaptomatrix. NMJs of galactose-1-phosphate uridyltransferase (*dGALT*)-null mutants exhibit marked changes in heparan sulfate proteoglycan co-receptor and Wnt ligand levels, which are effectively suppressed by the overexpression of *sgl*. The human orthologue of *sgl* is the human *UDP-glucose 6-dehydrogenase* (*UGDH*) gene, which is associated with EIEE (Table 1). Further studies with *Drosophila* models targeting *sgl* will be useful to clarify the link between the synaptomatrix carbohydrate environment and EIEE.

### 5.2. Drosophila Models for DEE other than EIEE

*Several Drosophila* models targeting genes associated with DEE other than EIEE have also been developed. They are summarized in the following sections.

#### 5.2.1. *Drosophila* Models Targeting Dynamin 1 like (DNM1L)

The *dynamin 1 like* (*DNM1L*) gene encodes dynamin-related protein 1 (DRP1), a GTPase of the dynamin superfamily. DRP1 plays an important role in mitochondrial and peroxisomal fission /division and in mitochondrial trafficking and distribution [177,178,179]. DRP1 contains three major dynamin domains called as the GTPase domain, the middle domain and the GTPase effector domain (GED). Pathogenic variants in DNM1L are associated with a mitochondrial encephalopathy with DEE. Individual patients have variable phenotypes ranging from severe hypotonia resulting in death in the neonatal period to developmental delay and regression. The patient carrying de novo heterozygous variant (Y691C) in the GED of *DNM1L* exhibits static encephalopathy, with a history of seizures and nystagmus [180].

*Drosophila* has a single homologue of *DNM1L* designated as *Drp1*. Transgenic flies carrying the human *DNM1L* gene or its mutant form *DNM1L^Y691C^* have been established [180]. *Drosophila drp1* mutants are lethal, exhibiting defects in mitochondrial trafficking to synapses, mitochondrial morphology and synaptic transmission [181]. Expression of human *DNM1L but* not the *DNM1L^Y691C^*, rescued the lethality of the *drp1* mutants [180]. Overexpression of *DNM1L^Y691C^* induced enlarged peroxisomes and abnormal perinuclear distribution, whereas that of wild-type protein exerted no effects on the peroxisomal morphology [180]. Overexpression of *DNM1L^Y691C^* in flight muscle induced a network of mitochondria near the muscle perinuclear region accompanied by the scarcity of mitochondria in muscle fibers, whereas that of wild-type protein did not, suggesting a dominant-negative effect of the mutant form [180]. These observations with the *Drosophila* model may explain the symptoms of the patient carrying the de novo heterozygous variant (Y691C).

#### 5.2.2. *Drosophila* Models Targeting Tmtc3

*Drosophila transmembrane O-mannosyltransferase targeting cadherins 3* (*Tmtc3*) encodes a mannosyltransferase that is predicted to be involved in protein O-linked mannosylation. It is orthologous to the human *transmembrane O-mannosyltransferase targeting cadherins 3* (*TMTC3*) gene. Human *TMTC3* is associated with an autosomal recessive neurologic disorder characterized by delayed psychomotor development, early-onset refractory seizures, intellectual disability with poor or absent speech and hypotonia. *Drosophila* models targeting *Tmtc3* have been established and characterized [182]. Neuron-specific knockdown of *Tmtc3* increased the bang-sensitivity, suggesting the increased susceptibility to mechanically induced seizures in *Drosophila* [182]. Of note, the increased bang-sensitivity was rescued by expression of the human *TMTC3* gene [182]. Detailed morphological analyses revealed that gross MB morphology, dendrite arborization in type 4 multidendrite neurons and synaptic morphology at NMJ were normal in the *Tmtc3* knockdown flies, suggesting functional defects in neuronal activity rather than the morphological defects in axons, dendrites and synapses [182]. As the human *TMTC3* gene is associated with a neuronal migration defect leading to Lissencephaly-8, further analyses with the *Drosophila* models targeting *Tmtc3* will be useful to gain more insight into this disorder.

#### 5.2.3. *Drosophila* Models Targeting Membrin

The *Drosophila membrin* gene encodes a protein that is predicted to have SNAP receptor activity and SNARE binding activity. Membrin is predicted to localize to the Golgi apparatus and cytoplasmic vesicles. It is a single orthologue of the human *Golgi SNAP receptor complex member 2* (*GOSR2*) that is associated with progressive myoclonic epilepsy (PME), a severe epilepsy syndrome characterized by childhood-onset myoclonus, ataxia, seizures and subsequent neurological decline. GOSR2 is an essential protein mediating ER to Golgi membrane fusion. Homozygous *Membrin* mutants die before 2nd instar larvae. *Drosophila* PME models targeting *Membrin* have been established by making transgenic flies carrying wild-type and mutant forms (G144W or K164del) of *Membrin* using site-specific ΦC31-mediated recombination to control for position effects on expression levels [183]. Transgenic flies carrying wild-type Membrin in the *Membrin* null background were viable and those carrying mutant Membrin were pharate adult lethal. Of note, ubiquitous overexpression of mutant Membrin in a wild-type background similarly caused pharate adult lethality. Neuron-specific expression of mutant Membrin also caused pharate adult lethality. *Drosophila* larvae carrying mutant forms of Membrin exhibited reductions in dendritic length and the terminal dendritic branch number. In addition, morphological defects in terminal synaptic boutons were detected. Furthermore, fluorescence recovery after photo-bleaching (FRAP) assay revealed that cargo trafficking activity in dendrites and synapse at NMJ were both reduced. Immunolabelling with the presynaptic active zone marker Bruchpilot (BRP) and postsynaptic GLURIII glutamate receptors also revealed the disruption of transsynaptic organization at NMJ in the *Drosophila* PME model. Furthermore, either fragmentation of the Futsch- and ANK2-XL-labeled cytoskeleton, which is normally continuous, or an absence of one or both of these proteins, were observed, suggesting a reduction of the local integrity of the presynaptic cytoskeleton. Electrophysiological assay revealed reduction in the frequency of spontaneous miniature excitatory postsynaptic potentials in larvae of the *Drosophila* PME models carrying mutant forms of Membrin. The *Drosophila* PME model larvae also had an increased duration of electrically induced seizures compared with the control.

Another group reported that ubiquitous knockdown of *Membrin* induced the seizure-like behavior in *Drosophila* adults characterized by twitching, wing flapping and loss of standing position after heating at 40 °C for 120 s [184]. Neuron-specific knockdown of *Membrin* using either the Elav-GAL4 or the nSyb-GAL4 driver induced no seizure-like behavior responding to heat, whereas a marked increase in heat-induced seizure-like behavior was observed with glia-specific knockdown of Membrin using the Repo-GAL4 driver [184]. Of note, the incidence of heat-induced seizure-like behavior increased with age. These observations suggest that Membrin is required exclusively in glial cells to prevent heat-induced seizure-like behavior during aging, although its requirement in neurons may not be excluded at the larval stage. The heat-induced seizure-like phenotype in adults was at least partially suppressed by treatment of sodium barbital, a GABA-agonist that is known to potently suppress seizures in human [184]. The *Drosophila* PME models will thus facilitate further studies on the pathophysiology of PMA, with the opportunity of identifying potential therapeutic targets.

#### 5.2.4. *Drosophila* Models Targeting Ube3a

*Drosophila ubiquitin protein ligase E3A* (*Ube3a*) encodes an E3 ubiquitin ligase transferring ubiquitin moieties from an E2 ligase to substrate proteins, which then targets them to be degraded by the ubiquitin proteasome system. *Drosophila* Ube3a contains a C-terminal HECT domain composed of 350 amino acids having high conservation with human UBE3A. *Ube3a* null mutants are viable but exhibit defects in climbing ability, the circadian wake and sleep rhythm and long-term associative olfactory memory [185]. The loss of Ube3a activity reduced the dendritic branching of sensory neurons in the peripheral nervous system.

Duplication 15q syndrome (Dup15q) is an autism-associated disorder concurrent with high rates of pediatric epilepsy that is caused by duplications of the chromosomal region 15q11.2-q13.1. Additional copies of the *UBE3A* gene are considered to induce Dup15q phenotypes. *Drosophila* models mimicking Dup15q have been established by overexpressing *Ube3a.* Glia-specific overexpression of *Ube3a* by repo-GAL4 increased bang sensitivity in adults, whereas neuron-specific overexpression by elav-GAL4 resulted in no bang-sensitive phenotype [186]. However, glia-specific overexpression of catalytically-inactive ligase-dead *Ube3a* exerted no effects on bang sensitivity, suggesting that the ubiquitin ligase activity of Ube3a is essential for bang sensitivity [186]. In addition, heat-induced and photogenic paralysis were observed in glia-specific *Ube3a*-overexpressing flies, suggesting that seizures can be initiated by multiple modalities [186]. Of note, glia-specific overexpression of human *UBE3A* also induced similar but less severe bang sensitivity. Immunostaining with anti-Fasciclin II antibody and anti-BRP antibody revealed abnormal development of neuroanatomical structures of MB. Glia-specific overexpression of *Ube3a* reduced the level of Na+/K+ pump ATPα, one of the substrates of Ube3a, thereby reducing the level of intracellular K+ within glial cells and increasing the concentration of K+ in the extracellular space, resulting the neuronal dysfunction and seizure behavior. *Drosophila* models targeting *Ube3a* are thus useful in characterizing Dup15q syndrome.

#### 5.2.5. *Drosophila* Models Targeting Prickle

The *Drosophila prickle* (*pk*) gene encodes a protein carrying PET and LIM domains. It is involved in the noncanonical Wnt signaling pathway to regulate intracellular calcium release and planar cell polarity. Its human orthologues are *prickle planar cell polarity protein 1* (*PRICKLE1*), *PRICKLE2 and PRICKLE3*. Among them, *PRICKLE1* is a causative gene for PME. Homozygous *pk^sple1^* mutants exhibit planar cell polarity abnormalities, including anomalies in the body epidermis and legs. Bang assay revealed that homozygous *pk^sple1^* mutants exhibit markedly delayed recovery that results in a prolonged climbing response compared with the control flies [187]. In addition, homozygous *pk^sple1^* mutant embryos have neuronal defects, including disorganization of the peripheral nervous system. Heterozygous *pk^sple1^* mutant flies were also bang-sensitive, even though they have no morphological defects. Other studies revealed that pk is responsible for organizing microtubule polarity and exerts effects on their growth dynamics in axons of *Drosophila* neurons, which consequently influences both anterograde and retrograde vesicle transport [188]. Bang-sensitive assay and electrophysiological assay revealed that the increase in the anterograde transport mechanism is mainly responsible for the seizure phenotype of the model flies, as it can be suppressed by reduction of the level of either of two Kinesin motor proteins responsible for anterograde vesicle transport such as Kinesin light chain and Kinesin heavy chain. *Drosophila* models targeting *pk* are thus useful for further characterizing PME.

### 5.3. Genetic Suppressors of Seizure Susceptibility in Drosophila

One of the advantages in using *Drosophila* models for epilepsy study is the use of classical and modern genetics to isolate suppressors of seizures. Suppressors of seizures may be novel targets for epilepsy therapy. For example, if suppressors have enzyme activity, their inhibitors can be good candidates for ASDs.

#### 5.3.1. Genetic Screening with Bang-Sensitive Mutants

Numerous seizure resistant mutants have been screened for their seizure suppressor activity and mutations in the *shaking B* (*shakB*), *para and maleless* (*mle*) genes have been identified [76]. *shakB* encodes a protein exhibiting gap junction channel activity. The loss-of-function *shakB* and *para* mutants with an evoked seizure threshold higher than wild-type effectively suppressed the seizure phenotype of several bang-sensitive mutants. The *mle* gene encodes an RNA helicase and is a member of the male-specific lethal dosage compensation complex that increases male-specific X chromosome transcription. It is orthologous to the human *DExH-box helicase 9* (*DHX9*) gene that is not related to epilepsy. The gain-of-function *mle* mutant with an evoked seizure threshold higher than that of wild-type also suppressed the seizure phenotype of several bang-sensitive mutants [76].

Mutagenesis by P-element random insertion in the genome under the *easily shocked* (*eas*) mutant genetic background was carried out to identify seizure suppressor genes [76]. The *eas* encodes an ethanolamine kinase involved in mechanosensory behavior, nervous system development and the phosphatidylethanolamine metabolic process, although its human orthologue *ethanolamine kinase 1* (*ETNK1*) is not related to epilepsy. The *eas* mutant exhibits a bang-sensitive paralytic phenotype. By this screen, nine seizure-suppressor mutations were identified, including the expected *para* and *shakB* alleles that validated the screening procedures. The others included *escargot* (*esg*), encoding a C2H2 zinc finger transcription factor involved in stem cell maintenance, tracheal morphogenesis and neuroblast differentiation, *meiotic P26* (*mei-P26*) encoding a protein involved in meiosis, germline differentiation and spermatogenesis, *topoisomerase 1* (*Top1*), encoding a topoisomerase with essential roles in cell proliferation during development and *Kazal-type protease inhibitor m1* (*Kaz-m1*), encoding a protein predicted to have serine-type endopeptidase inhibitor activity. Detailed analysis with the isolated *Top1* mutant revealed that seizure suppression is caused by P-element insertion in the 5′-UTR of the *Top1* gene, resulting in a reduction in *Top1* gene expression [189,190]. Although *Top1* is an essential gene, the isolated hypomorphic *Top1* allele is viable and commonly suppressed several bang-sensitive mutants. This suggested that reduced levels of Top1 activity in the mutants lead to DNA damage and cell death. Indeed, the *Top1* mutant had high levels of apoptosis in neurons and co-expression of DIAP1, an inhibitor of apoptosis, blocked suppression of the seizure phenotype of *eas*. Seizure suppressors like *Top1* therefore suggest novel classes of ASD targets with minimal side effects because they do not otherwise compromise nervous system function.

*Drosophila para^bss1^* mutants with a severe seizure phenotype were also used to identify and characterize seizure suppressor genes. As described above, *para^bss1^* mutants are highly bang-sensitive with a lower threshold for electric shock-induced seizures than wild-type. The *cac^TS2^* mutants effectively suppressed the seizure phenotype of the *para^bss1^* mutants [191]. Of note, the *para^bss1^* mutants reciprocally suppressed the seizure phenotype of the *cac^TS2^* mutants at a high temperature. Both genes are associated with EIEE as described above (Table 1). The *para* gene encodes a voltage-gated sodium channel α-subunit and the *cac* gene encodes the α1 subunit of a voltage-gated calcium channel. Based on these observations, mutations in different ion channels can form therapeutic combinations with effects that mask each other.

A set of chromosome deficiency lines were also utilized to screen the seizure suppressor or enhancer genes in the background of *para^bss1^* mutation [192]. Five chromosomal regions were identified as a deletion that can increase the bang-sensitive paralytic phenotype of *para^bss1^*. These deletion analyses followed by a screen of mutation of each gene located in the deletion identified the *charlatan* (*chn*) gene as a strong enhancer of the seizure phenotype. The *chn* gene encodes a C2H2 zinc-finger transcription factor involved in the development of sensory neurons, photoreceptors, blood cells, muscle and intestine, although there is no mammalian orthologue. Nine chromosomal regions were identified as deletions that can suppress the seizure phenotype of *para^bss1^*. The deletion analysis followed by genetic screening of mutations of each gene located in the deletion identified the *gilgamesh* (*gish*) gene as a strong suppressor of the seizure phenotype [192]. The *gish* gene encodes a plasma membrane-associated serine-threonine kinase that regulates Hedgehog, Wingless and Hippo signaling pathways. It is involved in planar cell polarity via the regulation of Rab11-mediated vesicle trafficking. It is orthologous to human *casein kinase 1 gamma 1* (*CSNK1G1*), *CSNK1G2 and CSNK1G3* genes. Seizure suppression by *gish* mutation may be specific to *para^bss1^* because it had no apparent effects on the seizure phenotype of *eas.* Moreover, the *gish* mutation increased the threshold for evoked seizures in *para^bss1^* mutants. Based on these studies with *Drosophila* models, the CSNK1G family may be a new target for seizure therapeutics.

Bang-sensitive assay and electrophysiology also identified the temperature-sensitive mutant *shi^ts1^* as having strong seizure suppressor activity [193]. The *shi^ts1^* is a temperature-sensitive missense mutant and mutant flies are paralyzed at the restrictive temperature [125,127]. The restrictive temperature required to reach complete paralysis for *shits* was unchanged in the presence of the bang-sensitive mutations such as *eas* and *para^bss1^*. However, the presence of *shits* mutations caused changes in response to mechanical stimulation, suggesting that they can act as suppressors of bang-sensitive mutants at the restrictive temperature. Increased temperature in the *shi^ts1^* mutant causes impaired synaptic vesicle recycling and is associated with suppression of the seizure-like activity. These observations suggest that targeting or limiting the availability of synaptic vesicles is an effective method to control epilepsy disorders.

#### 5.3.2. Genetic Screening with *Drosophila* Models Targeting Ube3a

Eye imaginal disc-specific overexpression of *Drosophila Ube3a* by the GMR-GAL4 driver induced morphological defects of the adult compound eye, the rough eye phenotype. The rough eye phenotype is a useful visible marker for extensive genetic screening, enabling the identification of genes that genetically interact with the target gene [75]. In many cases, the identified genes by this rough eye modifier screen are essential because they act as rate-limiting factors in the biological pathways related to the target genes. Three out of 346 deficiency lines were identified as enhancers of the rough eye phenotype induced by the overexpression of *Drosophila Ube3a*. Subsequently, three genes *IA-2*, *GABA-B-R3 and lola* (*longitudinals lacking*)*,* were identified as single genes responsible for the increased rough eye phenotype [194]. The *Drosophila IA-2* gene encodes a tyrosine phosphatase involved in gut development and insulin-like peptide secretion. It is orthologous to the human *protein tyrosine phosphatase receptor type N* (*PTPRN*) and *PTPRN2* genes. The *Drosophila GABA-B-R3* (*metabotropic GABA-B receptor subtype 3*) gene encodes a G-protein coupled receptor for the GABA neurotransmitter that is coupled to the Go G protein and activation of phospholipase C. Activation of the GABA-B-R3 can inhibit neuronal activity. Human orthologues of the other GABA_A_ receptor gene, *CG8916,* such as *GABRA1, GABRA2, GABRA5 and GABRG2,* are associated with EIEE (Table 1) as described above. The *Drosophila lola* gene encodes a putative transcription factor involved in Notch signaling, cell death, regulation of retrotransposons and transcriptional regulation of axon and dendrite patterning genes. There is no human orthologue of this gene. It may be of interest to examine the effects of mutations of these three genes on the epilepsy phenotype of *Ube3a*-overexpressing flies.

Another group investigated the gene regulatory network in which *Ube3a* is involved [195]. Mutations in *TCF4, MEF2C, UBE3A, ZEB2 or ATRX* cause phenotypically overlapping, syndromic forms of neurodevelopmental disorders with severe intellectual disability, epilepsy and microcephaly. Global or tissue specific knockdown or overexpression of each single orthologous *Drosophila* genes, such as *Da*, *Mef2*, *Ube3a*, *Zfh1* and *XNP and* their pairwise combinations were carried out to examine phenotypes such as lethality, wing and eye morphology, NMJ morphology, bang sensitivity and climbing behavior and then compared between single and pairwise dosage manipulations [195]. By these analyses, genetic interaction between *Ube3a* and *Mef2* via simultaneous dosage manipulation in tissues, such as glia, wing and eye, resulted in multiple phenotype alterations, although seizure susceptibility evaluated by the bang-sensitivity assay revealed no consistent phenotype [195]. These screening methods are applicable to any *Drosophila* epilepsy model and will advance the search of novel targets for epilepsy therapy.

### 5.4. Screening and Evaluation of ASDs with Drosophila Models

The *Drosophila* model is considered to be a powerful tool for screening of drugs, including ASDs [75]. In contrast to vertebrates, *Drosophila* has an open blood vascular system that enables drugs to be delivered to any target organ, including the brain [196]. There are two methods for the administration of drugs to flies. The first is the injection of drugs directly into the nervous system of *Drosophila*. The second, which is more convenient, is the feeding of flies by mixing drugs in the food or the preparation of filter paper soaked with a sucrose solution containing drugs. The scale of drug screening with *Drosophila* models is normally on the order of 100 to 500 small compounds per month, being smaller than the high-throughput screening of a large library of more than 100,000 small compounds in vitro or in cultured cells. However, in vivo screening with *Drosophila* models is expected to provide a high-quality hit.

#### 5.4.1. Evaluation of ASDs with Bang-Sensitive Mutants

Several known ASDs have been administered to *Drosophila* to evaluate their abilities to suppress seizures [76]. For chronic treatment, flies were raised in instant fly media containing a pre-determined specific dose of the drug, whereas for acute treatment, young adult flies were fed in a vial containing filter paper soaked with a sucrose solution containing a specific dose of the drug. In the bang assay with *eas* mutants, gabapentin reduced the average recovery time with chronic drug treatment but not with acute treatment [197]. Gabapentin increased the levels of the inhibitory neurotransmitter GABA, possibly through interaction with GABA metabolism or release. Gabapentin also inhibits calcium currents and may also affect sodium channels. Phenytoin is known to reduce both the average recovery time and the percentage of *eas* mutant flies exhibiting bang-sensitive behavior with both acute and chronic treatment [197]. Phenytoin binds to and blocks the inactive state of voltage-gated sodium channels. Phenytoin is also considered to block calcium channels and may increase the effects of GABA at certain receptor subtypes. Both gabapentin and phenytoin suppressed the seizure phenotype of *para^bss1^*. Carbamazeprine exerted no notable effects on the seizure phenotype of *eas* mutants, although it also blocked sodium channels in an activity-dependent manner. Ethosuximide, which blocks T-type Ca^2+^ channels and vigabatrin, which inhibits GABA transaminase to increase GABA levels, exerted no notable effects on the seizure phenotype of *eas* mutants [197].

Other studies revealed that potassium bromide involved in GABA potentiation and carbenoxolone involved in the inhibition of gap junctions are both effective to suppress the bang-sensitive phenotype in *Drosophila* models [76]. Furthermore, valproate inhibits voltage-gated Na+ channels and repetitious firing of action potentials to block T-type Ca^2+^ channels and to increase levels of GABA and GABA responses [76]. Other studies reported that injection of valproate into the brain or heart suppressed seizures in bang-sensitive mutants, although the extent of suppression was different depending on each mutant. In the feeding assay, valproate effectively suppressed seizure-like behaviors of *pk^sple1^* heterozygote flies [187]. However, in *para^bss1^* and *eas* mutants, feeding of valproate was not effective at ameliorating seizure phenotypes [189]. These reports suggest that many but not all, anticonvulsants used to treat human seizure disorders are also effective against *Drosophila* seizures [76].

#### 5.4.2. Screening of ASDs Focusing on the *Top1* Gene

Camptothecin (CPT) suppressed the seizure phenotype of the bang-sensitive mutants in the feeding assay. The *para^bss1^* mutants fed with CPT recovered from paralysis significantly faster than control flies and the tonic–clonic-like activity was almost completely suppressed [198]. CPT covalently binds to the Top1–DNA complex, thereby blocking re-ligation activity of Top1. Flavonoids with significant Top inhibitor activities, such as kaempferol and apigenin, also suppressed the seizure phenotype of the bang-sensitive mutants in the feeding assay. These observations suggest *Top1* to be a new target of ASD with minimal side effects [76].

#### 5.4.3. Evaluation of ASDs with the PTZ-Induced Kindling Epileptogenesis Model

Similar to rodents, the PTZ-induced kindling epileptogenesis model was developed in *Drosophila* [199]. Chronic PTZ treatment of *Drosophila* adults for 7 days induced a reduced climbing speed with an altered CNS transcriptome. Of note, this pattern of transcriptome was similar to gene expression alterations reported in human epileptogenesis. In this model, an increase in climbing speed is observed for 7 days after withdrawal of chronic PTZ. Administration of ethosuximide, gabapentin, vigabatrin, sodium valproate and levetiracetam after withdrawal of chronic PTZ recovered the decreased climbing speed of *Drosophila* model and normalized the transcriptomic perturbation in adult fly heads.

#### 5.4.4. Screening of ASDs Focusing on the *Pumilio* Gene

RNA sequence analyses with wild-type flies, *para^bss1^* mutants and wild-type flies fed with the proconvulsant picrotoxin revealed that 339 genes are consistently upregulated and 397 genes are downregulated in these seizure model flies [200]. The *pumilio* (*pum*) was found to be downregulated in both of these seizure models. The *pum* gene encodes a member of the PUF family of RNA-binding proteins that is also known as a homeostatic regulator of action potential firing in both *Drosophila* and mammals, regulating neuronal firing through binding to and regulating translation of the transcripts of voltage-gated sodium channels. It is orthologous to the human *pumilio RNA binding family member 1* (*PUM1*) and *PUM2* genes. Among them, the *PUM1* gene is associated with ataxia, developmental delay and epilepsy [201]. Of note, overexpression of *pum* in the cholinergic neuron of *para^bss1^* mutants suppressed the seizure phenotype, whereas its knockdown promoted it. It is well known that cholinergic neurons are the predominant excitatory interneurons in the insect CNS. Screening of a FDA-approved chemical library identified 12 compounds that increase *pum* promoter activity in cultured cells. Out of these 12 compounds, avobenzone effectively suppressed the seizure phenotype of *para^bss1^* mutants. The mode of action of avobenzone was demonstrated to include potentiation of *pum* gene expression that resulted in reduction of the persistent voltage-gated Na+ current in neurons.

Genome wide RNAi screen with cultured S2R+ cells identified 699 genes that increase *pum* gene promoter activity upon knockdown [202]. Among them, 101 genes are known to be regulated by synaptic activity. Fifty-seven of 101 genes suppressed the seizure phenotype of *para^bss1^* mutants when they were knocked down, suggesting that they are good candidates for ASD targets. Indeed, *para^bss1^* mutant larvae treated with inhibitors of these candidates, such as SB203580 (MAP kinase inhibitor), losmapimod (MAP kinase inhibitor), gemcitabine (ribonucleoside diphosphate reductase inhibitor), sodium fluoride (protein phosphatase inhibitor), metformin (NADH dehydrogenase inhibitor and commonly used to treat type II diabetes), bestatin (aminopeptidase inhibitor), WP1066 (JAK-STAT signaling inhibitor) or valproic acid (histone deacetylase inhibitor), similarly exhibited a significant reduction in seizure duration. Other studies also confirmed that metformin suppresses seizure-like activity in bang-sensitive mutants, including *eas* [89].

## 6. Perspectives

In the past decade, a series of genome-wide association studies and NGS identified a number of DEE-causing genes. In this review, we summarized recent studies with *Drosophila* models to clarify the in vivo roles of these genes especially focusing on EIEE-associated genes. However, successful development of suitable *Drosophila* models of EIEE-associate genes is limited and further efforts are needed. In addition to the classical mutants and the widely used GAL4-UAS targeted expression system combined with RNAi, introduction of CRISPR/Cas9 system to *Drosophila* in the past several years has enabled the development of more accurate *Drosophila* models for DEE carrying the same mutations found in patients. Elucidation of genetic variants of DEE using analogous mutations in the *Drosophila* genome will help to understand the molecular basis of the symptoms of EIEE.

Even after whole-genome sequencing, a substantial fraction of DEE cases remains unsolved. Possible causes of such unsolved cases include oligogenic inheritance and epigenetic abnormalities [203]. Inherited variants may lead to DEE through the synergistic effects of distinct deleterious variants involving common or distinct biological pathways [204]. Therefore, further studies are needed to investigate the roles of multiple gene interactions in DEE. *Drosophila* genetics is especially useful for analyzing these complex gene networks, which are important for understanding the genetic causes and pathophysiology of DEE. The combinatorial use of previously developed assays with *Drosophila* models, including the bang assay, NMJ analysis, dendrite arborization analysis, circadian wake and sleep rhythm analysis, learning and memory analysis and informatics-targeted screening, is promising. *Drosophila* is also useful for clarifying how environmental stress affects epigenetic regulators. It should be noted that gene ontology analysis identified 91 epigenetic regulators in *Drosophila and* many mutants and RNAi lines for these genes are available from the stock centers. In addition, chemical screening of FDA-approved drugs and natural compounds to develop a potential therapy for DEE will be more extensively carried out with *Drosophila* models. Although these studies with *Drosophila* models must be followed by assays using mammalian models and human, it is of interest whether the tested substances have therapeutic benefits for DEE patients.

## Figures and Tables

**Table 1:** Genes associated with early infantile epileptic encephalopathy (EIEE)

Encoded Gene Function			Gene Name (Human)	EIEE#	*Drosophila* Orthologs	Score
Ion channel	Sodium channel	Na_v_1.1	*SCN1A*	6	*para*	11 of 15
		Na_v_1.2	*SCN2A*	11		12 of 15
		Na_v_1.3	*SCN3A*	62		11 of 15
		Na_v_1.6	*SCN8A*	18		13 of 15
			*SCN1B*	52	*- (TipE/TEH1-4* *)	-
	Potassium channel	K_v_1.2	*KCNA2*	32	*Sh*	12 of 15
		K_v_2.1	*KCNB1*	26	*Shab*	9 of 15
		K_v_7.2	*KCNQ2*	7	*KCNQ*	10 of 15
		K_Ca_4.1	*KCNT1*	14	*SLO2*	13 of 15
		K_Ca_4.2	*KCNT2*	57		12 of 15
	HCN channel	HCN1	*HCN1*	24	*Ih*	6 of 15
	Calcium channel	Ca_v_2.1	*CACNA1A*	42	*cac*	10 of 15
		Ca_v_2.3	*CACNA1E*	69		10 of 15
Neurotransmitter receptor	GABA_A_ receptor		*GABRA1*	19	*CG8916*	10 of 15
			*GABRA2*	78		11 of 15
			*GABRA5*	79		9 of 15
			*GABRG2*	74		8 of 15
			*GABRB1*	45	*Lcch3*	14 of 15
			*GABRB3*	43		12 of 15
	GABA_B_ receptor		*GABBR2*	59	*GABA-B-R2*	14 of 15
	NMDA receptor		*GRIN2B*	27	*Nmdar2*	12 of 15
			*GRIN2D*	46		14 of 15
	AMPA receptor		*FRRS1L*	37	**-	-
Solute carrier family			*SLC1A2 (EAAT2)*	41	*Eaat2*	12 of 15
			*SLC12A5*	34	*kcc*	12 of 15
			*SLC13A5*	25	*Indy*	12 of 15
			*SLC25A22*	3	*GC1*	15 of 15
			*SLC25A12*	39	*aralar1*	15 of 15
			*SLC35A2*	22	*Ugalt*	13 of 15
Synaptic vesicle release/membrane trafficking			*DNM1*	31	*shi*	14 of 15
			*STXBP1*	4	*Rop*	15 of 15
			*CPLX1*	63	*cpx*	7 of 15
			*NECAP1*	21	*CG9132*	13 of 15
			*TRAK1*	68	*milt*	13 of 15
			*AP3B2*	48	*rb*	10 of 15
			*SYNJ1*	53	*Synj*	13 of 15
			*TBC1D24*	16	*sky*	15 of 15
Cell adhision molecule			*PCDH19*	9	**-	-
			*ADAM22*	61	*mmd*	8 of 15
Cytoskeletal protein			*SPTAN1*	5	*α-Spec*	15 of 15
			*PHACTR1*	70	*CG32264*	6 of 15
Intracellular signal transduction			*GNAO1*	17	*G* *αo*	15 of 15
			*CDKL5*	2	**-	-
			*RHOBTB2*	64	*RhoBTB*	13 of 15
			*PLCB1*	12	*Plc21C*	14 of 15
			*FGF12*	47	**-	-
			*SIK1*	30	**-	-
			*ARHGEF9*	8	**-	-
			*DOCK7*	23	*Zir*	15 of 15
			*DENND5A*	49	*pns*	14 of 15
			*SZT2*	18	**-	-
			*YWHAG*	56	*14-3-3ε*	15 of 15
			*NTRK2*	58	**-	-
			*CYFIP2*	65	*Sra-1*	15 of 15
			*DMXL2*	81	*Rbcn-3A*	11 of 15
Transcription factor			*ARX*	1	*al*	6 of 15
			*CUX2*	67	*ct*	8 of 15
			*NEUROD2*	72	**-	-
Translation			*EEF1A2*	33	*eEF1* *α2*	12 of 15
			*AARS*	29	*AlaRS*	14 of 15
			*PARS2*	75	*ProRS-m*	13 of 15
			*GUF1*	40	*waw*	13 of 15
Post-translational modification			*UBA5*	44	*Uba5*	15 of 15
			*RNF13*	73	*gzl*	10 of 15
			*ALG13*	36	*otu, CG14512*	8 of 15
			*PIGQ*	77	*PIG-Q*	6 of 15
			*PIGP*	55	*PIG-P*	9 of 15
			*PIGB*	80	*PIG-B*	13 of 15
			*PIGA*	20	*PIG-A*	15 of 15
Epigenetic factor			*ACTL6B*	76	*Bap55*	14 of 15
Mitochondrial enzyme			*MDH2*	51	*Mdh2*	14 of 15
			*GLS*	71	*GLS*	14 of 15
			*GOT2*	82	*Got2*	14 of 15
Others			*PNKP*	10	*CG9601*	13 of 15
			*ST3GAL3*	15	**-	-
			*CAD*	50	*r*	14 of 15
			*ITPA*	35	*CG8891*	15 of 15
			*HNRNPU*	54	*CG30122*	11 of 15
			*WWOX*	28	*Wwox*	13 of 15
			*ARV1*	38	*Arv1*	11 of 15
			*CNPY3*	60	*CNPYb*	10 of 15
			*PACS2*	66	*KrT95D*	13 of 15
			*UGP2*	83	*UGP*	12 of 15
			*UGDH*	84	*sgl*	15 of 15

Genes that recorded as EIEE-causative genes in Online Mendelian Inheritance in Man (OMIM) (https://omim.org/entry/308350#molecularGenetics) except for that of pending confirmation on April, 2020. *Drosophila* orthologs are referenced on FlyBase (https://flybase.org) and defined as genes, the scores presented on FlyBase are 6 point or higher out of 15 points. * There is no ortholog coding sodium channel beta subunit in *Drosophila*. *TipE* and *TEH1~4* are functional analogues.

**Table 2:** Comparisons of model animals and patient-derived induced pluripotent stem cell (iPSC).

Species	*Drosophila melanogaster*	*Caenorhabditis elegans*	*Danio rerio* (Zebrafish)	*Mus musculus* (Mice)	iPSC (Human)
Number of neurons	135,000	302	~1,000,000	71,000,000	-
Genome size	0.14 Gbp	0.10 Gbp	1.4 Gbp	2.8 Gbp	6.3 Gbp
Human disease genes conservative rate	75%	65%	82%	99%	100%
Generation time	10 days	4 days	3–4 months *	9–11 weeks	-
Complex behavior	+	+	++	+++	-

More number of + means more complex behavioral analysis is possible; -, not applicable. * Majority of zebrafish models are investigated up to 7 days.
